# Stroke Recognition for First Aid Providers: A Systematic Review and Meta-Analysis

**DOI:** 10.7759/cureus.11386

**Published:** 2020-11-08

**Authors:** Daniel Meyran, Pascal Cassan, Bert Avau, Eunice Singletary, David A Zideman

**Affiliations:** 1 Healthcare Division, French Red Cross, Paris, FRA; 2 Prehospital Emergency Care, Bataillon De Marins Pompiers De Marseille, Marseille, FRA; 3 International Federation of Red Cross and Red Crescent Societies (IFRC) Global First Aid Reference Center, French Red Cross, Paris, FRA; 4 Centre for Evidence-Based Practice, Rode Kruis-Vlaanderen, Mechelen, BEL; 5 Emergency Medicine, University of Virginia, Charlottesville, USA; 6 Pre-Hospital Emergency Medicine, Thames Valley Air Ambulance, Oxford, GBR

**Keywords:** stroke, score, prehospital, first aid, triage, recognition scale

## Abstract

Aim

To perform a systematic review of the literature on the effectiveness of existing stroke recognition scales used in a prehospital setting and suitable for use by first aid providers. The systematic review will be used to inform an update of international first aid guidelines.

Methods

We followed the Cochrane Handbook for Systematic Reviews of Interventions methodology and report results according to PRISMA guidelines. We searched Medline, Embase and CENTRAL on May 25, 2020 for studies of stroke recognition scales used by first aid providers, paramedics and nurses for adults with suspected acute stroke in a prehospital setting. Outcomes included change in time to treatment, initial recognition of stroke, survival and discharge with favorable neurologic status, and increased layperson recognition of the signs of stroke. Two investigators reviewed abstracts, extracted and assessed the data for risk of bias. The certainty of evidence was evaluated using GRADE methodology.

Results

We included 24 observational studies with 10,446 patients evaluating 10 stroke scales (SS). All evidence was of moderate to very low certainty. Use of the Kurashiki Prehospital SS (KPSS), Ontario Prehospital SS (OPSS) and Face Arm Speech Time SS (FAST) was associated with an increased number of suspected stroke patients arriving to a hospital within three hours and, for OPSS, a higher rate of thrombolytic therapy. The KPSS was associated with a decreased time from symptom onset to hospital arrival. Use of FAST Emergency Response (FASTER) was associated with decreased time from door to tomography and from symptom onset to treatment. The Los Angeles Prehospital Stroke Scale (LAPSS) was associated with an increased number of correct initial diagnoses. Meta-analysis found the summary estimate sensitivity of four scales ranged from 0.78 to 0.86. The FAST and Cincinnati Prehospital Stroke Scale (CPSS) were found to have a summary estimated sensitivity of 0.86, 95% CI [0.69-0.94] and 0.81, 95% CI [0.70-0.89], respectively.

Conclusion

Stroke recognition scales used in the prehospital first aid setting improves the recognition and diagnosis of stroke, thereby aiding the emergency services to triage stroke victims directly down an appropriate stroke care pathway. Of those prehospital scales evaluated by more than a single study, FAST and Melbourne Ambulance Stroke Screen (MASS) were found to be the most sensitive for stroke recognition, while the CPSS had higher specificity. When blood glucose cannot be measured, the simplicity of FAST and CPSS makes these particular stroke scales appropriate for non-medical first aid providers.

## Introduction

Stroke is one of the leading causes of death and disability worldwide [1]. The early detection of stroke in the prehospital setting has the potential to improve stroke outcomes by decreasing delays in treatment. A variety of stroke assessment scales have been developed for both in-hospital and prehospital use. Stroke scales designed for the prehospital setting have a lower number of diagnostic criteria, easy-to-identify clinical signs and simplicity of implementation, making them applicable for use by first aid providers and lay persons. In 2015, the International Liaison Committee on Resuscitation (ILCOR) published a Consensus on Science with Treatment Recommendations (CoSTR), suggesting a benefit from the first aid use of stroke recognition scoring systems or scales for individuals with suspected acute stroke [2, 3].

The objective of this systematic review was to synthesize the evidence for the diagnostic accuracy and clinical effectiveness of stroke scales applied by laypeople, paramedics and nurses in a prehospital setting, according to the research question: Among adults with suspected acute stroke, does the use of a rapid stroke scoring system or scale, compared with basic first aid assessment without the use of a scale, change time to treatment, recognition of stroke, discharge with favorable neurologic status, survival with favorable neurologic outcomes, and increase the public/layperson recognition of stroke signs?

## Materials and methods

This systematic review was conducted in accordance with the Cochrane Handbook for Systematic Reviews of Interventions [4], and reporting occurred through the Preferred Reporting Items for Systematic Reviews and Meta-Analyses (PRISMA) checklist [5]. This review will inform the International Liaison Committee on Resuscitation (ILCOR) consensus on science and treatment recommendations for stroke recognition.

Eligibility criteria and outcomes

The population included adults over 18 years old, suspected of having a stroke in the prehospital setting, regardless of its type or severity, including ischemic stroke, hemorrhagic stroke or transient ischemic attack (TIA). We excluded all patients with trauma.

The intervention/index test was the use of a single, rapid stroke scale during primary patient assessment to diagnose stroke, as used by a first aid provider, paramedic or nurse. We excluded studies where stroke scales were applied in an emergency department, or assessments made by general practitioners or neurologists. We also excluded stroke scales intended to assess for large vessel occlusion as these were felt to be beyond the skill of a lay first aid provider.

Comparison groups included suspected stroke patients, managed by first aid providers, paramedics or nurses in the prehospital setting who did not use a stroke scale during the primary assessment. To measure the diagnostic accuracy of stroke scales, studies compared the stroke scale result to the hospital diagnosis of stroke as a reference test. An in-hospital diagnosis of stroke was a confirmed documented physician or imaging diagnosis.

The critical outcome was the time to treatment. This outcome included the proportion of patients whose time from symptom onset to hospital arrival or treatment was within two or three hours, time from symptom onset to arrival in the emergency department or hospital, time between hospital arrival to computed tomography (CT) head scan or other imaging (‘door’ to imaging) and time from symptom onset to administration of tissue Plasminogen Activator (tPA) or the use of endovascular reperfusion techniques.

For the important outcome of recognition of stroke, two types of data studies were eligible: clinical efficacy studies, assessing the proportion of patients receiving appropriate treatment, and diagnostic accuracy studies. Other important outcomes were discharge with favorable neurologic status, survival with favorable neurologic outcome, and cognitive knowledge. The latter outcome evaluated whether stroke recognition scales improve first aid provider recognition of signs of stroke.

Study designs

Randomized controlled trials (RCTs) and non-randomized studies (non-randomized controlled trials, interrupted time series, controlled before-and-after studies, cohort studies, diagnostic test accuracy studies) were eligible for inclusion. Unpublished studies, conference abstracts, trial protocols and posters were excluded. All languages were included as long as there was an English abstract.

Information sources and search strategy

We included studies from the 2015 International Liaison Committee on Resuscitation (ILCOR) consensus on first aid science with treatment recommendations (CoSTR) systematic review of stroke assessment scales [2, 3]. The existing search strategy, previously run from inception through January 15, 2015, was re-run in MEDLINE (PUBMED interface), EMBASE (Embase interface), and the Cochrane Central Register of Controlled Trials (CENTRAL) from January 1, 2014 to September 29, 2019 (Appendix A). The search was re-run on May 25, 2020. Additional studies were identified through a hand search of reference lists from included studies.

After removal of duplicates, two authors (PC, DM) independently screened titles and abstracts for relevance. Full texts of potentially relevant publications were retrieved and evaluated by the same reviewers, independently. Papers judged to be relevant were included and reasons for exclusion were documented. Discrepancies between the reviewers were resolved by discussion with the ILCOR First Aid Task Force. Inter-rater reliability was measured with Cohen’s kappa at the title and abstract stage and the full text article stage [6].

Data collection

We used a prespecified data extraction form to collect the following data from included studies: number of participants, age, study characteristics (study design, country, inclusion and exclusion criteria), intervention, training method, reference standard for diagnostic studies, outcome measures and findings. Where possible, missing values were calculated from the available data. For diagnostic studies, we extracted 2 × 2 data (true positives, false positives, true negatives and false negatives) directly for each index test.

Risk of bias and certainty of evidence assessment

For observational studies, the risk of bias (ROB) and certainty of evidence for each individual study was assessed using the Risk Of Bias In Non-randomized Studies of Interventions (ROBINS-I) tool [7]. For diagnostic studies, we assessed the risk of bias of each study using the Quality Assessment of Diagnostic Accuracy Studies version 2 (QUADAS-2) tool [8]. A study was considered at high risk of bias if one of the domains within the ROBINS-I tool or QUADAS-2 tool identified high risk of bias. The Grading of Recommendations Assessment, Development and Evaluation (GRADE) methodology was used to determine the certainty of evidence for the body of evidence across outcomes [9]. In the GRADE approach, the certainty of evidence can be high, moderate, low or very low. Observational studies assessed with the ROBINS-I tool and diagnostic test accuracy studies assessed with the QUADAS-2 tool start with a high level of certainty [7, 10, 11] and can be downgraded across five domains (limitations in study design, imprecision, indirectness, heterogeneity and publication bias), and upgraded across three domains (large magnitude of effect, dose-response and residual plausible bias and confounding).

Data analysis

Continuous outcomes are reported as mean differences (MD) with 95% confidence intervals (CIs). Dichotomous outcomes are reported as risk ratios (RR) with 95% CIs. There was insufficient data to conduct meta-analyses of effectiveness data. For diagnostic studies, all scales used the same positivity threshold of ‘one or greater’, which indicates that the person was considered to have a stroke with one or more positive criteria. For each index test, we generated a diagnostic 2 × 2 table (true positives, false positives, true negatives and false negatives) from which we calculated sensitivity and specificity with 95% confidence intervals (CI). When more than one study was identified per scale, we calculated a summary point estimated sensitivity and specificity using a random effects meta-analysis and created Summary Receiver Operating Characteristic (SROC) plots to show the variation in test accuracy estimates across studies with Review Manager 5.3 (RevMan 5.3, The Nordic Cochrane Centre, Copenhagen, Denmark, 2014). Parameter values required by Review Manager Software to construct plots in the SROC space were calculated with MetaDTA: Diagnostic Test Accuracy Meta-Analysis website, version 1.25 (https://crsu.shinyapps.io/dta_ma/) [12].

## Results

**Figure 1:**
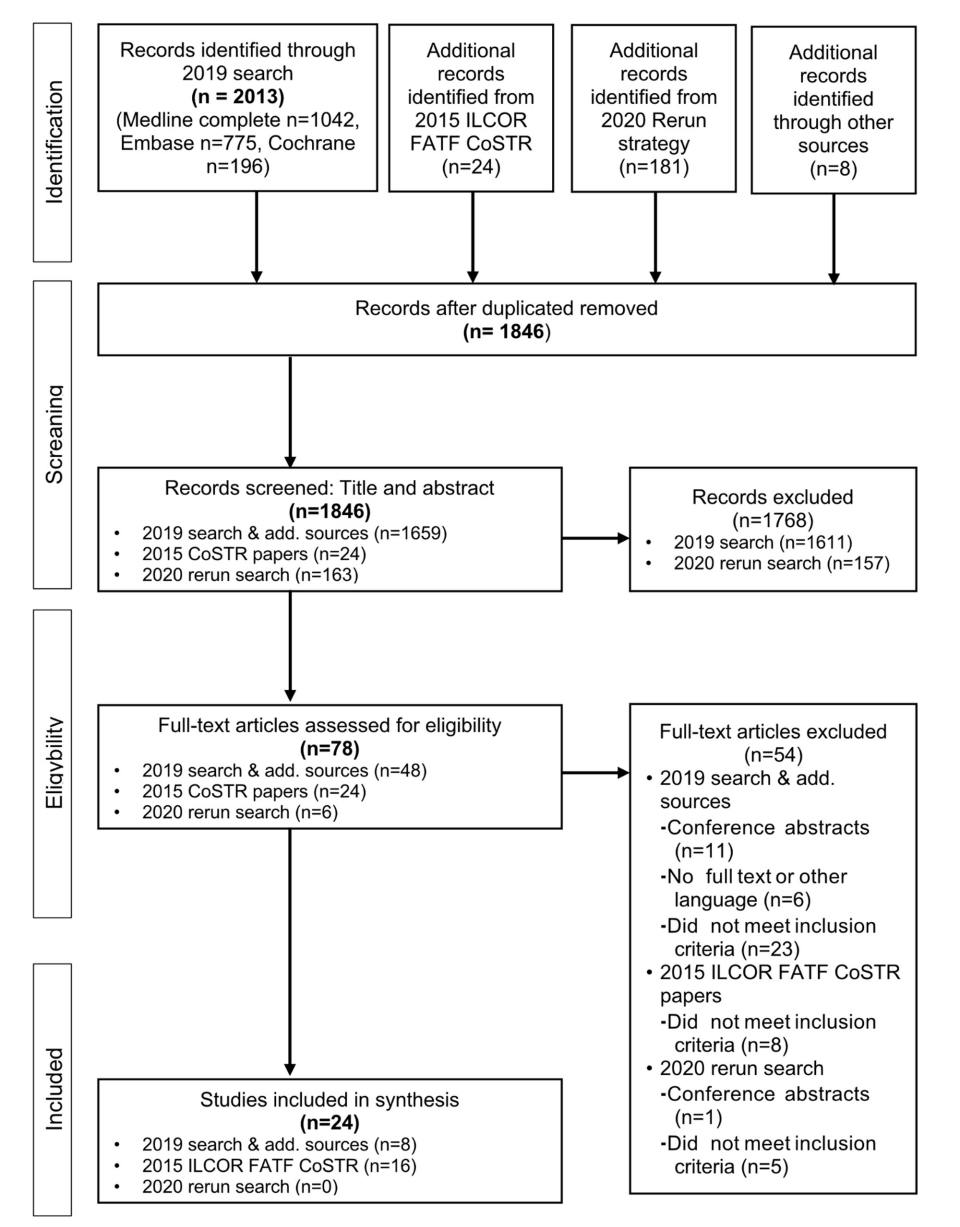
PRISMA diagram (diagram illustrating the flow of articles throughout the selection procedures) CoSTR: Consensus on Science with Treatment Recommendations; FATF: First Aid Task Force; ILCOR: International Liaison Committee on Resuscitation; PRISMA: Preferred reporting items for Systematic Reviews and Meta-Analyses.

For the literature search and study selection, an updated search strategy from 2014 to 2019 and a rerun search strategy from 2019 to 2020 identified 1814 unique titles/abstracts. In addition, we identified new studies and 24 from the previous 2015 search results for the 2015 ILCOR CoSTR for first aid stroke assessment [2]. Based on title and abstract screening, we excluded 1768 studies (reviewer agreement was 95.15%, Kappa = 0.44). Of the 78 full-text articles reviewed, a further 54 were excluded (reviewer agreement was 99.87%, Kappa = 0.79). We ultimately included a total of 24 studies (Figure 1).

Study characteristics

Characteristics of the included studies are summarized in Table 1. Excluded studies with reasons are presented in Appendix B. We included 24 observational studies; 13 were prospective [13-25], and 11 were retrospectives studies [26-36]. Five studies assessed time to treatment or recognition of stroke outcomes [20, 23, 24, 32, 34], 18 studies assessed the diagnostic accuracy of stroke recognition scales [13-22, 25-28, 30-32, 35, 36] and one study assessed both time to treatment and diagnostic accuracy [29]. Four studies investigated the “Face, Arm, Speech, Time (FAST)” scale [14, 17, 21, 27], five studies investigated the “Los Angeles Prehospital Stroke Scale (LAPSS)” [14-16, 18, 26], 12 studies investigated the “Cincinnati Prehospital Stroke Scale (CPSS)” [14, 15, 19, 22, 25, 26, 28, 30-32, 35, 36], and three studies investigated the “Melbourne Ambulance Stroke Screen (MASS)” scale [14, 15, 28]. The “Face, Arm, Speech, Time, Emergency Response Protocol (FASTER)” scale, “Ontario Prehospital Stroke Scale (OPSS)”, “Kurashiki Prehospital Stroke Scale (KPSS)”, “Recognition of Stroke in the Emergency Room (ROSIER)” scale, “Medic Prehospital Assessment for Code Stroke (MedPACS)”, “Balance, eyes, FAST (BeFAST)” and “Prehospital Ambulance Stroke Test (Pre-HAST)” were investigated by one study each [13, 17, 20, 21, 29, 34, 36]. One study investigated education in stroke signs and symptoms [23]. Sixteen studies investigated only one scale [13, 16, 18-20, 22, 24, 25, 27, 29-35] and seven studies investigated two or more scales [14, 15, 17, 21, 26, 28, 36]. The characteristics of stroke recognition scales evaluated in these studies are described in Table 2.

**Table 1:** Characteristics of published meta-analyses ACLS: Advanced Cardiac Life Support; CPSS: Cincinnati Prehospital Stroke Scale; CT: Computerised tomography; DVD: Digital Versatile Disc; ED: Emergency Department; EEG: Electroencephalogram; EMD: Emergency Medical Dispatcher; EMS: Emergency Medical Service; EMT: Emergency Medical Technician; FAST: Face Arm Speech Time; FASTER: Face, Arm, Speech, Time, Emergency Response; GCS: Glasgow Coma Scale; ICD: International Classification of Diseases; ICH: Intracerebral Haemorrhage; IV: Intravenous; KPSS: Kurashiki Prehospital Stroke Scale; LAPSS: Los Angeles Prehospital Stroke Scale; MASS: Melbourne Ambulance Stroke Screen; MedPACS: Medic Prehospital Assessment for Code Stroke; MRA: Magnetic Resonance Angiography; MRI: Magnetic Resonance Imaging; NIHSS: National Institute of Health Stroke Score; OPSS: Ontario Prehospital Stroke Scale; PreHAST: PreHospital Ambulance Stroke Test; ROSIER: Recognition of Stroke in the Emergency Room; TIA: Transient Ischemic Attack; tPA: Tissue plasminogen activator.

Study (Author, year)	Study design	Population description	Inclusion/exclusion criteria	Scales	Reference standard use	Test administrator	Training	Outcomes
Andsberg et al. (2017) [13]	Prospective observational study	Hässleholm, Sweden. N = 69, mean age not reported.	Inclusion: suspicion of stroke, deﬁned as sudden onset of focal neurologic symptoms/signs, in conscious people > 18 years of age.	PreHAST	After reviewing medical records by two stroke physicians.	Ambulance nurses	Four-hour education program including practical training under supervision and proper execution.	Diagnostic accuracy
Asimos et al. (2014) [26]	Retrospective observational, cross-sectional study	North Carolina, US. N = 2442. Mean age = 66 years (CPSS) and 69 years (LAPSS). 25.2% men.	Inclusion: preliminary EMS impression of stroke. Exclusion: patients with duplicate data records and patients who were transferred between facilities.	CPSS, LAPSS	ED diagnosis of stroke, used ICD 9/10 codes without any other detail.	Paramedics	Not reported	Diagnostic accuracy
Bergs et al. (2010) [14]	Prospective observational cross-sectional study	Leuven, Belgium. N = 135. Mean age > 77 years. 61% men.	Inclusion: all adults transported with relevant neurologic complaints. Exclusion: ages < 18 years, GCS < 9, transported to alternate hospital, trauma, form t filled.	FAST, CPSS, LAPSS, MASS	Unspecified, diagnosis at ED discharge.	Emergency nurses	Briefing on purpose of study, stroke scales and guidelines	Diagnostic accuracy
Berglund et al. (2014) [27]	Retrospective observational study	Stockholm, Sweden. N = 900. Range age = 22-93 years. 55.5% men.	Inclusion: all persons from 18 to 85 years suspected of having a stroke with onset within six hours and with independence in activities of daily living.	FAST	Diagnosis of stroke after imaging, neurologic exam, EEG, laboratory tests. All participants received a final diagnosis by a neurologist or stroke specialist.	Paramedics	One lecture about stroke about the FAST test prior to the start of the study.	Diagnostic accuracy
Bray et al. (2005) [15]	Prospective observational cross-sectional study	Melbourne, Australia. N = 100.	Inclusion: preliminary EMS impression of stroke or suspicion of stroke by dispatchers. Exclusion: not reported.	CPSS, LAPSS, MASS	Diagnosis of stroke at discharge (stroke/TIA registry)	Paramedics	One-hour educational session, and instruction in assessment and documentation of items used in a prehospital stroke scale.	Diagnostic accuracy
Bray et al. (2010) [28]	Retrospective observational Study	Melbourne, Australia. N = 850.	Inclusion: patients with suspicion of stroke and TIA. Exclusion: patients who were unconscious or asymptomatic at the time of paramedic assessment.	CPSS, MASS	Stroke/TIA registry to determine if the discharge diagnosis was stroke or TIA.	Paramedics	One-hour stroke education program and instruction in the use of MASS.	Diagnostic accuracy
Chen et al. (2013) [16]	Prospective observational study	Beijing, China. N = 1130. Age ranges = 20-101 years, median 72 years. 60.5% men.	Inclusion: patients suspected of stroke and TIA. Absence of coma. Exclusion: patients < 18 years, unconscious, trauma and no neurological complaints.	LAPSS	Discharge diagnosis of stroke (clinical diagnosis).	Paramedics	180 min training station with three experts from study team.	Diagnostic accuracy
Chenkin et al. (2009) [29]	Retrospective observational study	Toronto, Canada. N = 325.	Inclusions: symptoms suggesting an acute neurologic problem. Exclusion: patients with stroke mimic, patients needing emergent intervention and patient’s ineligibility for fibrinolysis, terminally ill or palliative.	OPSS	Final in-hospital diagnosis of acute stroke defined as either ischemic stroke, ICH or TIA according to the consulting neurologist.	Paramedics.	90-minute training session on stroke screening tool prior to implementation.	Diagnostic accuracy. tPA administration rates before and after implementation of the protocol. Prehospital transport intervals.
English et al. (2018) [30]	Retrospective observational study	Rochester, Michigan, US. N = 130. Mean age = stroke 76.6 years, stroke 72.1 years. 50% men.	Inclusion: stroke suspected in adults by EMS in the field. Exclusion: hospital arrival via helicopter; outside hospital transfer; direct admission without ED evaluation and last known well time greater than 6 hours.	CPSS	Final diagnosis documented at discharge.	Paramedics	One-hour online module annually on stroke recognition and assessment.	Diagnostic accuracy. Time from EMS dispatch to arrival on scene. On-scene time. Transport time.
Fothergill et al. (2013) [17]	Prospective observational study	London, United Kingdom. N = 295. Mean age = 65 years, range 20-95 years; 53% men.	Inclusion: patients of age > 18 years presenting with symptoms of stroke. Exclusion: age < 18 years, patients without ROSIER scale in assessment or transfer to another hospital.	FAST, ROSIER	Final diagnosis of stroke, TIA or non-stroke made by medical physicians with CT and MRI scans (clinical team to confirm).	Paramedics	One-hour stroke educational program, scenario-based demonstration of ROSIER and 15-minute educational DVD.	Diagnostic accuracy
Frendl et al. (2009) [31]	Retrospective observational study	Durham, United State. N = 154. Mean age = 67 years. 44% men.	Inclusion: all participants transported by EMS and having possible stroke or TIA. Exclusion: unresponsive patient.	CPSS	Participants’ final diagnosis in the hospital stroke registry (clinical, laboratory and radiographic evaluations).	Paramedics	One-hour interactive educational presentation on stroke recognition and use of the CPSS.	Diagnostic accuracy. On scene time (min).
Greenberg et al. (2017) [32]	Retrospective observational study	Philadelphia, US. N = 305. Mean age = 66 years. 50.8% men.	Inclusion: all patients seen with the admitting diagnosis of stroke and onset of symptoms was < 6 hours.	CPSS	Final diagnosis documented at discharge.	Paramedics	Training courses on CPSS during ACLS training.	Diagnostic accuracy. Door to CT time. Door to physician time. Door to needle (administration of tPA) time.
Harbison et al. (2003) [33]	Retrospective observational study.	Newcastle, Bournemouth, United Kingdom. N = 487. Mean age = 72 years.	Inclusion: Stroke/TIA suspected patients, GCS > 7. Exclusion: subarachnoid haemorrhage.	FAST	Final discharge diagnoses based on the results of clinical assessment and imaging (Following six months).	Paramedics	Training package (lecture notes, slide presentation, handout, and multiple choice questionnaire) presented to ambulance staff and newly recruited staff.	Diagnostic accuracy
Iguchi et al. (2010) [34]	Retrospective observational study	Kurashiki city, Japan. N = 30. Mean age = 73 years. 61.9% men.	Inclusion: consecutive patients transferred to hospital by paramedics finally diagnostic as having an acute stroke or TIA within 24 h of onset.	KPSS	Stroke or TIA was diagnostic based on the results of imaging, MRA and carotid duplex ultrasonography immediately after admission.	Paramedics	90-min training session	Symptom onset to admission time between 0 and 3 hours. Intravenous tPA. Neurologic manifestation. Rate of IV-tPA. Correlation between KPSS (paramedics) and NIHSS (neurologist) after excluding patients with onset > 3 hours before admission.
Kidwell et al. (2000) [18]	Prospective observational study	Los Angeles, US. N = 206. Mean age = 63 years. 52% male.	Inclusion: non-comatose, non-trauma suspected strokes in adults (people with neurologically relevant symptoms). Exclusion: asymptomatic upon EMS arrival, age < 18 years.	LAPSS	Final diagnosis of stroke at hospital after a review of reports, imaging and physician notes.	Paramedics	One-hour initial training session with video and a LAPSS certification.	Diagnostic accuracy
Kim et al. (2017) [19]	Prospective observational study	Busan, Republic of Korea. N = 268.	Inclusion: patients with suspected stroke, patients who were transported to hospital by paramedics and patients with true stroke admitted during the same period.	CPSS	Final diagnosis of stroke or TIA (no other mention).	Paramedics	Not reported	Diagnostic accuracy
Kothari et al. (1999) [25]	Prospective observational study	Cincinnati, United states. N = 171. Mean age = 57.8 years. 72% men.	Inclusion: patients with stroke, TIA, a stroke-mimicking condition, or a combination of these conditions or patients with other neurologic disorders recruit in an ED service and neurology service.	CPSS	CPSS made by physician.	Paramedics	10-minute review on how to perform CPSS with paramedics and EMTs. Only verbal instructions were given.	Diagnostic accuracy
O’Brien et al. (2012) [20]	Prospective observational study	Gosford, Australia. N = 115.	Inclusion: all patients with an initial diagnostic of acute stroke.	FASTER	Not reported.	Paramedics	Information about implementation FAST protocol.	Proportion of ischemic stroke patients who received tPA. Symptom onset to hospital arrival. ED door-to-CT scan. ED door-to-needle (tPA administration). ED door-to-Stroke Unit. Adverse events.
Pickham et al. (2019) [21]	Prospective observational study	Santa Clara County (California), US. N = 359.	Inclusion: patients with sudden onset of neurological symptoms < 6 hours from EMS arrival were assessed. Exclusion: patients presenting directly to the ED.	FAST, BEFAST	The patient’s final diagnosis based on chart review by experienced stroke nurses at each participating hospital.	Paramedics	One-hour training video.	Diagnostic accuracy
Ramanujam et al. (2008) [35]	Retrospective observational study	San Diego, United states. N = 1045.	Inclusion: patient with acute stroke identification by EMD or paramedics and age > 18 years. Exclusion: patients who were taken to other acute care hospitals, not transported by City EMS agency or with no final outcome data.	CPSS	Stroke team diagnostic or hospital discharge diagnostic.	Paramedics	Not reported	Diagnostic accuracy
Studnek et al. (2013) [36]	Retrospective observational study	Charlotte, North Carolina. N = 416. Mean average age = 66.8 years. 45.7% male.	Inclusion: suspected stroke or TIA patients who received a prehospital MedPACS screen and were transported to one of the seven local hospitals. Exclusion: age < 18 years, unconscious, seizures, no documented assessment, secondary transports.	CPSS, MedPACS	Stroke diagnosis at hospital discharge.	Nurses	2-hour continuing education lecture regarding neurologic emergencies.	Diagnostic accuracy
Vanni et al. (2011) [22]	Prospective observational study	Firenze, Roma, and Pescara, Italy. N = 155. Mean age = 72 years. 59% men.	Inclusion: presence at triage of acute focal neurological deficits or a local EMS dispatch for suspected stroke. Exclusion: major trauma and coma (GCS < 8). Patients with terminal illnesses (life expectancy < 3 months).	CPSS	Stroke diagnoses were established by a consensus of three experts after reviewing all clinical data and imaging results.	Nurses	Not reported.	Diagnostic accuracy
Wall et al. (2008) [23]	Prospective observational study	Massachusetts, Boston, United states. Age = 40 to 64 years.	Inclusion: Women from the Well-Integrated Screening and Evaluation for Women Across the Nation (WISEWOMAN).	FAST	None	Lay public	Education session with 3-minute animation to teach the signs of stroke.	Knowledge changes immediately after 3-month training.
Wojner-Alexandrov et al. (2005) [24]	Prospective observational study	Houston, United states. N = 446. Mean age = 69 years. 44% male.	Inclusion: stroke suspected in adults by the dispatcher or EMS provider in the field. Exclusion: none.	LAPSS	Final discharge diagnostic (definitive diagnostic determined by neurologist).	Paramedics	Monthly paramedic education based on Brain Attack Coalition and American Stroke Association.	Diagnostic accuracy. Time to symptom onset to ED arrival. Paramedic transport times. Time to ED arrival to CT interpretation. Treatment with intravenous tPA.

**Table 2:** Characteristics of prehospital stroke recognition scales BEFAST: Balance Eyes Face Arm Speech Time on call; CPSS: Cincinnati Prehospital Stroke Scale; FAST: Face Arm Speech Time; FASTER: Face, Arm, Speech, Time, Emergency Response; KPSS: Kurashiki Prehospital Stroke Scale; LAPSS: Los Angeles Prehospital Stroke Scale; MASS: Melbourne Ambulance Stroke Screen; MedPACS: Medic Prehospital Assessment for Code Stroke; OPSS: Ontario PreHospital Stroke Scale; PreHAST: PreHospital Ambulance Stroke Test; ROSIER: Recognition of Stroke in the Emergency Room. 1. Verbal instruction and sensory, Close your eyes! Grip your hand! (n-paretic side); 2. GCS < 7 or suspected head injury exclusion original paper; 3. Seizure at onset, can be transported to arrive within two hours of onset, time since symptom onset < 2 hours, GCS < 10, blood glucose > 4 mmol/L, symptoms of the stroke have resolved; 4. Blood glucose > 3.5 mmol/L, history of seizure; 5. History of seizure, time since symptom onset < 24 hours, at baseline, patient is not wheelchair bound or bedridden, age > 45 years, blood glucose 2.8 to 22.2 mmol/L; 6. History of seizure, time since symptom onset < 24 hours, at baseline, patient is not wheelchair bound or bedridden, blood glucose 3.3 to 22.2 mmol/L; 7. History of seizure, at baseline, patient is not wheelchair bound or bedridden, blood glucose 2.8 to 22.2 mmol/L, age limit = 40 years; 8. Age > 18 years, intended for use, only in conscious people, i.e. alert or aroused by stimulation; 9. Time of onset less than 2 hours, blood glucose measurement inside the range of 4-17 mmol/L.

Assessment	FAST	CPSS	OPSS	KPSS	ROSIER	MASS	Med PACS	LAPSS	PreHAST	FASTER	BEFAST
Number of physical examination items	3	3	4	5	5	4	5	3	8	5	5
Facial droop	Yes	Yes	Yes		Yes	Yes	Yes	Yes	Yes	Yes	Yes
Arm weakness/drift	Yes	Yes	Yes	Yes	Yes	Yes	Yes	Yes	Yes	Yes	Yes
Leg weakness/drift			Yes	Yes	Yes		Yes		Yes		
Hand grip strength						Yes		Yes			
Stability										Yes	
Speech difficulty	Yes	Yes	Yes	Yes	Yes	Yes	Yes		Yes	Yes	Yes
Eye position, gaze preference							Yes		Yes		
Visual field					Yes				Yes	Yes	
Eye diplopia											Yes
Sensory (pain)									Yes		
Balance coordination											Yes
Command, verbal instruction									Yes^1^		
Consciousness disturbance				Yes							
Level of consciousness				Yes							
Score range	0-3	0-3	0-4	0-13	-2 to 5	0-4	0-5	0-3	0-19	0-5	0-5
Eligibility criteria	Yes^2^		Yes^3^		Yes^4^	Yes^5^	Yes^6^	Yes^7^	Yes^8^	Yes^9^	Yes
Blood glucose measurement			Yes		Yes	Yes	Yes	Yes		Yes	

Risk of bias within studies and certainty of the evidence

An overview of the assessment of the overall certainty of evidence, using ROBINS-I assessment tool for non-randomized studies of interventions studies and QUADAS-2 for diagnostic studies is provided in Tables 3, 4 respectively. Overall, the certainty of evidence was moderate to very low across all outcomes, primarily due to risk of bias, indirectness and imprecision. A detailed overview of GRADE assessments per outcome can be found in Appendix C.

**Table 3:** Risk of bias in non-randomized studies of interventions (ROBINS-I)

	Domain							
Study (Author, year)	Confounding	Selection	Classification of intervention	Deviation from intended intervention	Missing data	Outcomes	Selective reporting	Overall
Chenkin et al. (2009) [29]	Serious	Low	Low	Serious	Serious	Low	Low	Very serious
Harbison et al. (2003) [33]	Information	Low	Serious	Low	Low	Low	Low	Very serious
Iguchi et al. (2011) [34]	Low	Serious	Low	Low	Serious	Moderate	Low	Very serious
Wojner-Alexandrov et al. (2005) [24]	Low	Serious	Low	Low	Low	Low	Low	Serious
O'Brien et al. (2012) [20]	Serious	Serious	Low	Low	Low	Serious	Low	Very serious
Wall et al. (2008) [23]	Low	Low	Low	Low	Low	Low	Low	Low

**Table 4:** Certainty assessment of diagnostic accuracy studies (QUADAS 2)

	Risk of bias				Applicability concerns		
Study (Author, year)	Patient selection	Index test	Reference standard	Flow and timing	Patient selection	Index test	Reference standard
Andsberg et al. (2017) [13]	Low	Low	Low	Low	Low	Low	Low
Asimos et al. (2014) [26]	High	Low	High	Low	Low	Low	Low
Bergs et al. (2010) [14]	High	Low	Unclear	Unclear	Low	Low	Low
Bray et al. (2005) [15]	High	Low	Unclear	Unclear	Low	Low	Low
Berglund et al. (2014) [27]	Low	Low	Low	Low	Low	Low	Low
Bray et al. (2010) [28]	High	Low	Unclear	Unclear	Low	Low	Low
Chen et al. (2013) [16]	High	Low	Low	Unclear	Low	Low	Low
Chenkin et al. (2009) [29]	High	Low	Unclear	Unclear	Low	Low	Low
English et al. (2018) [30]	High	Low	Unclear	Unclear	Low	Low	Low
Fothergill et al. (2013) [17]	High	Low	Unclear	Low	Low	Low	Low
Frendl et al. (2009) [31]	High	Low	Unclear	Unclear	Low	Low	Low
Greenberg et al. (2017) [32]	Low	Low	Low	High	Low	Low	Low
Kothari et al. (1999) [25]	Unclear	Low	Low	Low	Low	Low	Low
Kidwell et al. (2000) [18]	Low	Low	Low	Unclear	Low	Low	Low
Kim et al. (2017) [19]	High	Low	Unclear	Unclear	Low	Low	Low
Pickham et al. (2019) [21]	High	Low	High	Low	Low	Low	Low
Ramanujam et al. (2008) [35]	High	Low	Unclear	Unclear	Low	Low	Low
Studnek et al. (2013) [36]	High	Low	Unclear	Unclear	Low	Low	Low
Vanni et al. (2011) [22]	Low	Low	Low	Low	Low	Low	High

Study findings on stroke assessment scale effectiveness

For the critical outcome “time to treatment”, we identified four observational studies [20, 24, 29, 34] evaluating four different stroke scales (KPSS, LAPSS, OPSS, FASTER). For the KPSS, one retrospective observational study [34], enrolling 430 participants with suspected acute stroke in the prehospital setting, showed an association between the use of KPSS and an increase in the number of patients whose time from symptom onset to hospital arrival was within 3 hours. Of patients who had the KPSS applied, 161/256 (62.9%) arrived within 3 hours compared with 91/174 (52.3%) who did not have the scale applied (RR 1.2; 95% CI [1.01 - 1.43]; p = 0.034: very low certainty evidence). The same study showed significantly shorter elapsed time from symptom onset to hospital admission with the use of KPSS (mean time 2.1 hours; interquartile range [1.0 - 6.2]), compared with no KPSS use (mean time 2.7 hours; interquartile range [1.2 - 9.7]; p = 0.024; very low certainty evidence). For the LAPSS, one observational study [24], enrolling 1518 prehospital participants with suspected acute stroke, showed an association between the use of the LAPSS and an increased time from symptom onset to emergency department arrival (MD 132.00 min; 95% CI [14.68 - 249.32]; p = 0.097; very low certainty evidence). The same study did not find a significant benefit associated between use of LAPSS and the proportion of patients admitted within 120 min (RR 1.07; 95% CI [0.96 - 1.19]; p = 0.215; very low certainty evidence). For OPSS, one observational study [29], enrolling 861 prehospital participants with acute suspected stroke, showed an association between use of the OPSS and increased proportion of patients with a time from symptom onset to hospital arrival within 3 hours when using the OPSS, compared with not using the OPSS (RR 1.43; 95% CI [1.12 - 1.82]; p = 0.004; very low certainty evidence). For FASTER, one observational study [20], enrolling 115 prehospital participants, showed an association between use of FASTER and a shortened time from symptom onset to treatment with tissue Plasminogen Activator (tPA) (MD -32 min; 95% CI [-53 to -11]; p = 0.005; very low certainty of evidence). Furthermore, this study showed an association between the use of FASTER and a shorter door to CT time for patients receiving tPA (MD -30 min; 95% CI [-49 to -11] p = 0.004, very low certainty of evidence), and a shorter “door to needle” time for patients receiving tPA (MD -46 min; 95% CI [-71 to -21] p = 0.001, very low certainty of evidence). Among patients receiving tPA, no significant differences were found between the groups with or without FASTER applied for time from symptom onset to hospital arrival (MD, 17 min; 95% CI [-7 to 41]; p = 0.180, very low certainty of evidence). We did not identify any comparative studies evaluating the other scales (FAST, ROSIER, MASS, CPSS, MedPACS and PreHAST) for the critical outcome “time to treatment”.

For the important outcome “recognition of stroke” (outcome defined as definitive stroke diagnosis or therapy administration), we identified five observational studies [20, 24, 29, 33, 34] evaluating five different stroke scales (FAST, KPSS, FASTER, OPSS, LAPSS). For the FAST scale, one observational study [33], enrolling 356 prehospital participants with suspected acute stroke, showed an association with use of FAST and an increased proportion of patients with confirmed stroke or TIA admitted within 3 hours following symptom onset (RR 3.3; 95% CI [2.29 - 4.75]; p < 0.00001, low certainty evidence). For KPSS, one observational study [34], enrolling 430 prehospital participants with suspected acute stroke, showed no difference between use and non-use of KPSS for the proportion of patients who were diagnosed with stroke and received thrombolytic therapy (RR 0.95; 95% CI [0.59 - 1.53]; p = 0.838, low certainty evidence). For LAPSS, one observational study [24], enrolling 1518 prehospital participants, showed an association with the use of LAPSS by paramedics and an increased proportion of correct initial diagnoses of stroke as confirmed by a neurologist (RR 1.29; 95% CI [1.18 - 1.42]; p < 0.00001, moderate certainty evidence). However, no association was found with the use of the LAPSS and the proportion of patients treated with intravenous tPA among confirmed stroke cases (RR 1.13; 95% CI [0.71 - 1.80]; p = 0.601, moderate certainty evidence). For OPSS, one observational study [29], enrolling 861 prehospital participants, showed no association between the use of OPSS and the rate of recognition of ischemic stroke (RR 1.11; 95% CI [0.96 - 1.28]; p = 0.157, low certainty evidence), but did show an association between the use of OPSS and an increased rate of thrombolytic therapy in ischemic stroke cases (RR 1.72; 95% CI [1.03 - 2.88]; p = 0.037, low certainty evidence). For FASTER, one observational study [20], including 182 participants, showed an association between the use of FASTER and an increased proportion of stroke patients who received thrombolytic therapy (RR 2.56; 95% CI [1.02 - 6.45]; p = 0.045, very low certainty evidence).

For the important outcome of increased public/layperson recognition of stroke signs, one observational study [23], enrolling 72 participants (members of the public), was included. This study reported that immediately after training compared with pre-training, there was a significant increase in the percentage of participants who recognized facial droop, arm weakness and slurred speech as signs of stroke (68/72 (94.4%) compared with 55/72 (76.4%); RR 1.24; 95% CI [1.07 - 1.42]; p = 0.003, moderate certainty evidence). Of the 65 participants who were retested three months after the training, compared with pre-training, 100% remembered slurred speech and facial drooping as stroke symptoms; 98.5% remembered arm weakness or numbness, showing no significant change from the immediate post-training test (moderate certainty of evidence).

We did not identify any comparative studies evaluating stroke recognition for the outcomes of “favorable neurologic status” or “survival with favorable neurologic outcome”.

**Figure 2:**
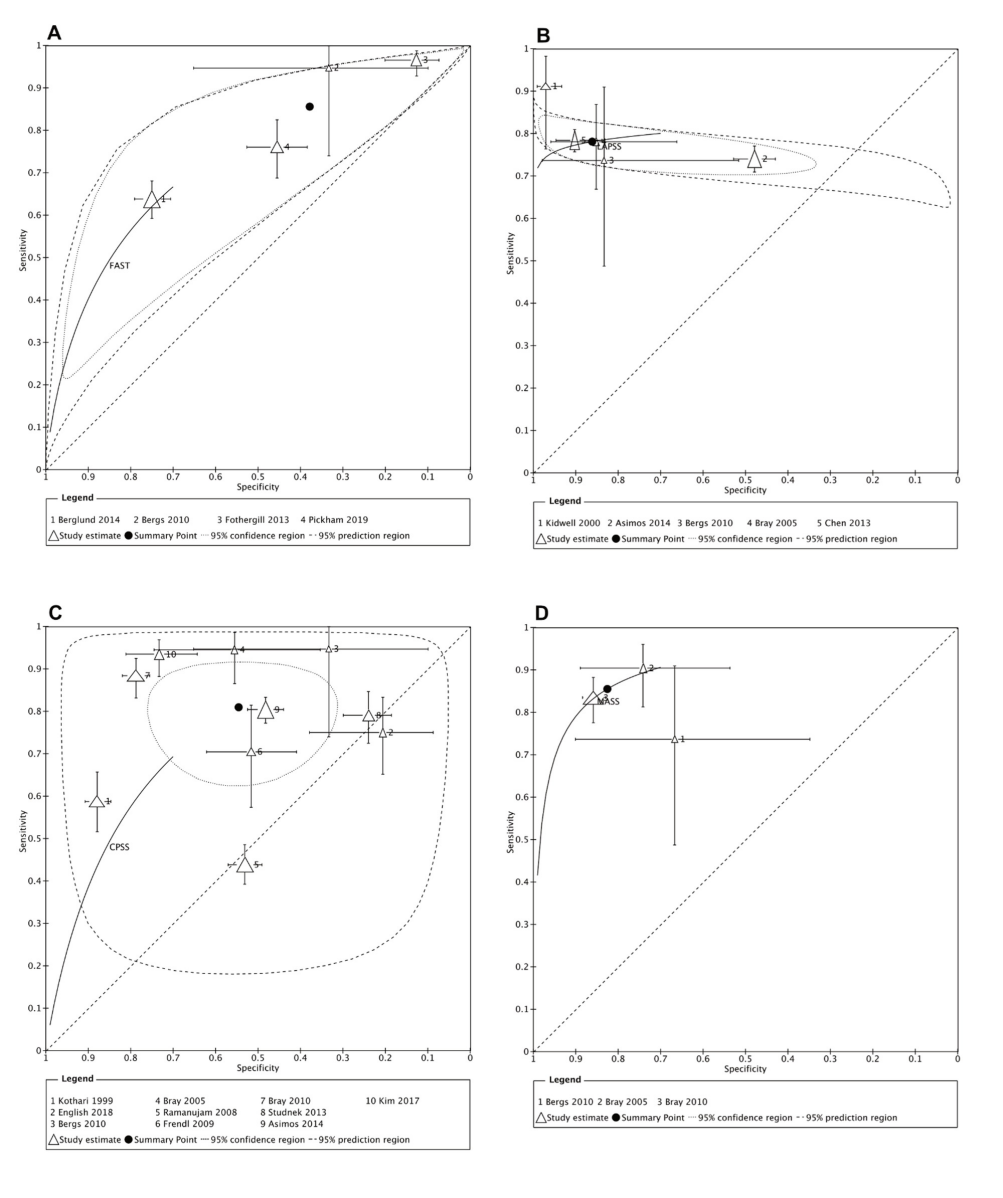
Summary receiver operating characteristics plots A- Face Arm Speech Time (FAST); B- Los Angeles Prehospital Stroke Scale (LAPSS); C- Cincinnati Prehospital Stroke Scale (CPSS); D- Melbourne Ambulance Stroke Scale (MASS).

For the outcome of recognition of stroke (diagnostic studies, outcome defined as correct stroke diagnosis), we identified 19 observational studies [13-19, 21, 22, 25-32, 35, 36] including a total of 8153 participants, evaluating nine different screening tools (FAST, LAPSS, OPSS, CPSS, ROSIER, MASS, BEFAST, Med-PACS, Pre-HAST) (Table 5). The reported prevalence, sensitivity, specificity, positive and negative likelihood ratio for each scale are reported in Table 5. Four scales, FAST (Figure 2A), LAPSS (Figure 2B), CPSS (Figure 2C) and MASS (Figure 2D), were assessed by more than one study. The diagnostic accuracy of the FAST scale was assessed by very low certainty evidence from four observational prospective studies [14, 17, 21, 27], including 1585 participants suspected of having a stroke. The summary estimate for sensitivity was 0.86, 95% CI [0.69-0.94] and the summary estimate for specificity was 0.38, 95% CI [0.16-0.66]. The diagnostic accuracy of the LAPSS was assessed by low certainty evidence from four prospective observational studies [14-16, 18] and one retrospective study [26]. The studies included a total of 2692 participants suspected of having a stroke. The summary estimate for sensitivity was 0.78, 95% CI [0.75-0.81] and the summary estimated diagnostic specificity was 0.86, 95% CI [0.67-0.95]. The diagnostic accuracy of the CPSS was assessed by very low certainty evidence from six prospective observational studies [14, 15, 19, 22, 25, 28] and six retrospective observational studies [26, 30-32, 35, 36]. The studies included a total of 4842 participants suspected of having a stroke. The summary estimate for sensitivity was 0.81, 95% CI [0.70-0.89] and the summary estimate for specificity was 0.55, 95% CI [0.39-0.69]. Two additional studies were identified [22, 32], but these provided incomplete data and could not be included in the meta-analysis. The diagnostic accuracy of the MASS was assessed by low certainty evidence from two prospective observational studies [14, 15] and one retrospective observational study [28]. These three studies included a total of 981 participants suspected of having a stroke. The summary estimate for sensitivity was 0.85, 95% CI [0.79-0.90] and the summary estimate for specificity was 0.82, 95% CI [0.69-0.91].

**Table 5:** Operating characteristics of prehospital stroke scales by included study FAST: Face Arm Speech Time; CPSS: Cincinnati Prehospital Stroke Scale; LAPSS: Los Angeles Prehospital Stroke Scale; MASS: Melbourne Ambulance Stroke Screen; Med PACS: Medic Prehospital Assessment for Code Stroke; OPSS: Ontario PreHospital Stroke Scale; ROSIER: Recognition of Stroke in the Emergency Room; PreHAST: PreHospital Ambulance Stroke Test; BEFAST: Balance Eyes Face Arm Speech Time on call.

Stroke Scale	Study (Author, year)	Sample size	Stroke prevalence (Number/total, %)	Sensitivity (95% CI)	Specificity (95% CI)	Positive likelihood-ratio (95% CI)	Negative likelihood-ratio (95% CI)
FAST	Bergs et al. (2010) [14]	31	19/31 (61%)	0.95 [0.74-1.00]	0.33 [0.10-0.65]	1.42 [0.94-2.15]	0.16 [0.02-1.25]
	Fothergill et al. (2013) [17]	295	177/295 (60%)	0.97 [0.93-0.99]	0.13 [0.07-0.20]	1.11 [1.03-1.19]	0.27 [0.11-0.67]
	Berglund et al. (2014) [27]	900	472/900 (52%)	0.64 [0.59-0.68]	0.75 [0.71-0.79]	2.55 [2.14-3.05]	0.48 [0.42-0.55]
	Pickham et al. (2019) [21]	359	159/359 (44%)	0.76 [0.69-0.82]	0.46 [0.38-0.53]	1.40 [1.20-1.63]	0.53 [0.38-0.72]
CPSS	Asimos et al. (2014) [26]	1217	663/1217 (54%)	0.80 [0.77-0.83]	0.48 [0.44-0.52]	1.55 [1.42-1.70]	0.41 [0.35–0.48]
	Bergs et al. (2010) [14]	31	19/31 (61%)	0.95 [0.74-1.00]	0.33 [0.10-0.65]	1.42 [0.94-2.15]	0.16 [0.02-1.25]
	Bray et al. (2010) [28]	850	199/850 (23%)	0.88 [0.83-0.93]	0.79 [0.75-0.82]	4.17 [3.57-4.88]	0.15 [0.10-0.22]
	Bray et al. (2005) [15]	100	73/100 (73%)	0.95 [0.87-0.98]	0.56 [0.35-0.75]	2.13 [1.39-3.25]	0.10 [0.04-0.27]
	Frendl et al. (2009) [31]	154	61/154 (40%)	0.70 [0.57-0.81]	0.52 [0.41-0.62]	1.46 [1.12-1.90]	0.57 [0.37-0.88]
	Kothari et al. (1999) [25]	171	49/171 (29%)	0.59 [0.52-0.66]	0.88 [0.85-0.91]	4.88 [3.74-6.37]	0.47 [0.40-0.55]
	Ramanujam et al. (2008) [35]	1045	440/1045 (42%)	0.44 [0.39-0.49]	0.53 [0.49-0.57]	0.93 [0.82-1.07]	1.06 [0.95-1.18]
	English et al. (2018) [30]	130	96/130 (74%)	0.75 [0.65-0.83]	0.21 [0.09-0.38]	0.94 [0.77-1.16]	1.21 [0.58-2.56]
	Kim et al. (2017) [19]	268	152/268 (57%)	0.93 [0.88-0.97]	0.73 [0.64-0.81]	3.50 [2.58-4.74]	0.09 [0.07-0.17]
	Studnek et al. (2013) [36]	416	186/416 (45%)	0.79 [0.72-0.85]	0.24 [0.19-0.30]	1.04 [0.94-1.15]	0.88 [0.61-1.26]
	Vanni et al. (2011) [22]	155	87/155 (56%)	Not estimated	Not estimated	Not estimated	Not estimated
	Greenberg et al. (2017) [32]	305	79 (26%)	Not estimated	Not estimated	Not estimated	Not estimated
LAPSS	Asimos et al. (2014) [26]	1225	805/1225 (66%)	0.74 [0.71-0.77]	0.48 [0.43-0.53]	1.42 [1.28-1.57]	0.54 [0.47-0.63]
	Bergs et al. (2010) [14]	31	19/31 (61%)	0.74 [0.49-0.91]	0.83 [0.52-0.98]	4.42 [1.21-16.12]	0.32 [0.14-0.70]
	Bray et al. (2005) [15]	100	73/100 (73%)	0.78 [0.67-0.87]	0.85 [0.66-0.96]	5.27 [2.12-13.13]	0.26 [0.16-0.41]
	Chen et al. (2013) [16]	1130	997/1130 (88%)	0.78 [0.76-0.81]	0.90 [0.84-0.95]	8.02 [4.78-13.46]	0.24 [0.21-0.27]
	Kidwell et al. (2000) [18]	206	34/206 (16%)	0.91 [0.76-0.98]	0.97 [0.93-0.99]	31.36 [13.14-74.87]	0.09 [0.03-0.27]
MASS	Bergs et al. (2010) [14]	31	19/31 (61%)	0.74 [0.49-0.91]	0.67 [0.35-0.90]	2.21 [0.95-5.14]	0.39 [0.17-0.93]
	Bray et al. (2010) [28]	850	199/850 (23.4%)	0.83 [0.78-0.88]	0.86 [0.83-0.88]	5.90 [4.84-7.20]	0.19 [0.14-0.26]
	Bray et al. (2005) [15]	100	73/100 (73%)	0.90 [0.81-0.96]	0.74 [0.54-0.89]	3.49 [1.84-6.63]	0.13 [0.06-0.27]
Med PACS	Studnek et al. (2013) [36]	416	186/416 (45%)	0.74 [0.67-0.80]	0.33 [0.27-0.39]	1.10 [0.97-1.25]	0.79 [0.58-1.08]
OPSS	Chenkin et al. (2009) [29]	554	214/554 (39%)	0.87 [0.82-0.92]	0.59 [0.54-0.65]	2.15 [1.87-2.47]	0.21 [0.15-0.31]
ROSIER	Fothergill et al. (2013) [17]	295	177/295 (60%)	0.97 [0.93-0.99]	0.18 [0.11-0.26]	1.18 [1.08-1.28]	0.19 [0.08-0.46]
PreHAST	Andsberg et al. (2017) [13]	69	26/69 (38%)	1.00 [0.87-1.00]	0.40 [0.25-0.56]	1.65 [1.30-2.11]	0.00
BEFAST	Pickham et al. (2019) [21]	359	159/359 (44%)	0.91 [0.86-0.95]	0.26 [0.20-0.33]	1.23 [1.12-1.36]	0.34 [0.19-0.59]

**Figure 3:**
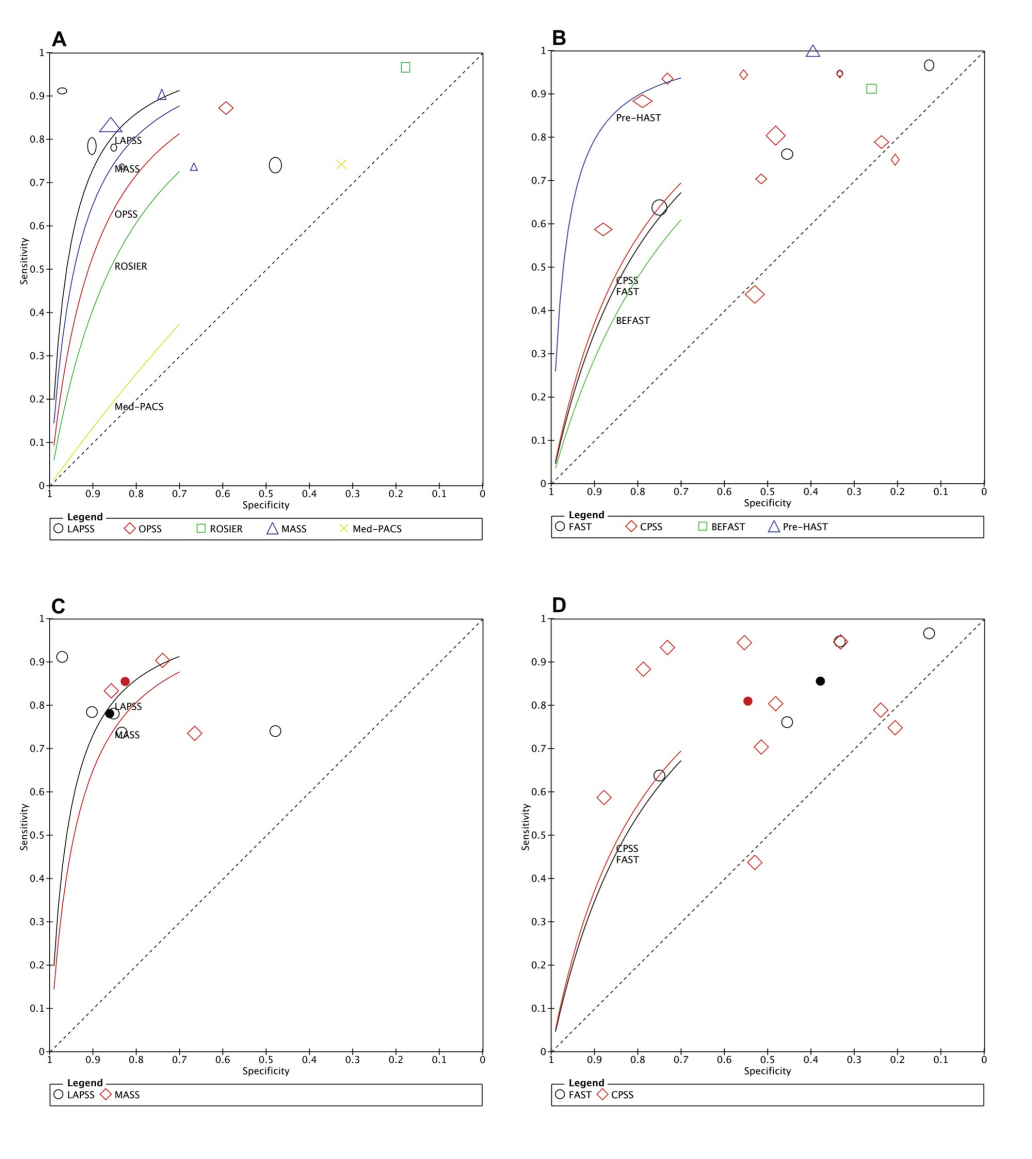
Summary receiver operating characteristics (SROC) plot of strokes scales with and without glucose measurement A- SROC of stroke scales with glucose measurement; B- SROC of stroke scales without glucose measurement; C- SROC of stroke scales with more than one study per scale with glucose measurement; D- SROC of stroke scales with more than one study per scale without glucose measurement.

Studies of stroke assessment scales can be divided into subgroups based on whether the scale includes blood glucose measurement or not. In the nine diagnostic studies that used stroke scales with blood glucose measurement (LAPSS, OPSS, ROSIER, MASS, Med-PACS) [14-18, 26, 28, 29, 36], the reported sensitivities ranged from 0.74 to 0.97, compared with 0.80 to 1.00 in the 14 studies of stroke scales that did not include blood glucose measurement (FAST, CPSS, Pre-HAST, BEFAST) [13-15, 17, 19, 21, 25-28, 30, 31, 35, 36]. The reported specificities from studies with stroke scales including blood glucose measurement (LAPSS, OPSS, ROSIER, MASS, Med-PACS) ranged between 0.18 and 0.86 compared with 0.26 to 0.55 in the studies that used scales without blood glucose measurement (PreHAST, FAST, CPSS, BEFAST). The comparison of Summary Receiver Operating Characteristics (SROC) curve between stroke scales with blood glucose measurement and stroke scales without blood glucose measurement is presented in Figure 3. The first comparison covers all studies (Figure 3A, 3B); the second covers only the scores assessed by more than one study (Figure 3C, 3D).

## Discussion

We identified and systematically reviewed studies of accuracy for prehospital stroke recognition tools that are applied in the prehospital setting and potentially suitable for use by first aid providers. We consider an ideal stroke assessment tool for first aid to be one that is easily understood and remembered, has a high sensitivity for detecting stroke and can be completed in minimal time. Because the home use of blood glucose measurement devices is increasingly common in populations at risk for acute stroke, we included prehospital stroke scales that incorporate blood glucose measurements but evaluated them separately for accuracy. In this systematic review, three of the four included scales (KPSS, FASTER and OPSS) showed an association between prehospital use and a decreased time from stroke onset to treatment [20, 29, 34]. Unfortunately, it was not possible to perform a meta-analysis for this outcome due to the limited number of studies.

In terms of definitive stroke diagnosis or therapy administration, using a stroke recognition scale in the prehospital setting does not seem to increase the proportion of patients with confirmed stroke diagnosis. However, patients with confirmed stroke were promptly admitted to a hospital and received treatment more quickly.

For accuracy of recognition of stroke we pooled the data from the 17 diagnostic studies of FAST, CPSS, LAPSS, and MASS individually to calculate a summary estimated sensitivity and specificity [14-19, 21, 22, 25-28, 30-32, 35, 36]. Other scales that were only assessed by a single study were not included [13, 17, 21, 29, 36]. We considered both the FAST and CPSS to be stroke assessment tools that a first aid provider would find easy to understand, remember and to use. These two stroke scales are supported by multiple studies with a large total number of participants but do not include a blood glucose measurement. For FAST, the sensitivities in four studies ranged from 0.64 to 0.97 [14, 17, 21, 27] with a summary estimated sensitivity of 0.86 [0.69-0.94]. For CPSS, the sensitivity measurements from 10 included studies ranged from 0.44 to 0.97 [14, 15, 19, 25, 26, 28, 30, 31, 35, 36] with a summary estimated sensitivity of 0.78 [0.75-0.81]. Specificities of these two scales were significantly less, ranging from 0.13 to 0.88, with a lower summary estimated specificity for FAST compared with CPSS (0.38 [0.16-0.66] compared with 0.86 [0.67-0.95]). Thus, whilst FAST appears to be a more sensitive scale for the recognition of stroke, it is less specific than the CPSS. The high risk of bias and between-study heterogeneity did not allow us to determine summary estimates sensitivity and specificity of scales with and without blood glucose measurement. Some studies reported a high specificity of above 0.90, where the proportion of missed stroke patients assessed by the scale ranged from 10 to 22% [16, 18]. Prehospital stroke recognition scales should not be interpreted as confirmatory diagnostic tests but only as a screening test. Most of the studies only assessed the outcomes of true positive patients, however it would be of value to know the impact of the scale on those who were false negatives.

Two stroke assessment scales that include blood glucose measurement in their eligibility criteria (MASS and LAPSS) were evaluated by multiple studies and included 981 patients for MASS and 2692 patients for LAPSS (Table 5). We found these scales had similar sensitivities for stroke identification as for scales without blood glucose measurement, but increased specificities (Figure 3C, 3D). We recognize that many first aid providers may not have access or the skills to use a properly calibrated glucometer. Local guidelines would need to determine the benefit of increased specificity of stroke scales that include glucose measurement compared with using simpler stroke scales that do not require glucose measurement.

Three systematic reviews analyzed stroke recognition instruments in the prehospital setting [37-39]. Brandler et al. in 2014 included studies in which the scales were used by paramedics or emergency medical technicians (EMTs) and included scales requiring blood glucose measurement [37]. The authors concluded that LAPSS performed more consistently and that LAPSS and CPSS had similar diagnostic capabilities. Our systematic review includes all of the studies evaluated by Brandler et al. and adds new data from 16 more publications. Additionally, we report diagnostic accuracy of scales that require blood glucose measurement separately from those without glucose measurement, to help identify appropriate scales for use by first aid providers. A systematic review by Rudd et al. in 2016 included all studies in which the scales were administered face-to-face by any prehospital or hospital clinician to identify adults suspected of stroke [38]. Eleven studies included in this systematic review were also included in our review, but 10 studies did not meet our inclusion criteria (seven papers and three abstracts). The authors concluded that available data do not allow a strong recommendation to be made about the superiority of a particular stroke recognition scale evaluated. Zhelev et al. in a Cochrane review in 2019 analyzed prehospital stroke scales as screening tools for early identification of stroke and transient ischemic attack [39]. They included in a “prehospital setting subgroup” all studies where the scale had been used in the prehospital setting regardless of the background and training of the person performing the assessment, and only evaluated diagnostic accuracy. The author concluded, “in the field, CPSS had consistently the highest sensitivity but was less specific than most of the scales”. In our systematic review, we have focused on the scales that can potentially be used by trained first aid providers or lay persons in a prehospital setting. We attribute our inclusion and exclusion criteria to any differences in our results. Lastly, our systematic review is not limited to a diagnostic accuracy review. We also evaluated the influence of stroke scale use on the time to treatment and the rate of stroke diagnosis.

Our review has some limitations. First, only four stroke scales were investigated by more than a single study, for which a large number of participants have been included (FAST, CPSS, LAPSS, MASS). Six scales (FASTER, OPSS, KPSS, ROSIER, BEFAST, Med-PACS) were only investigated in single studies, including between 250 and 600 participants [13, 17, 20, 21, 29, 34, 36]. The PreHAST scale provided the highest sensitivity (1.00, 95% CI [0.87-1.00]), but was only evaluated in a single study, with 69 participants [13]. The prevalence of stroke/TIA ranged from 23% [28] to 88% [16] (Table 5), reflecting differences in population and patient selection that may affect sensitivity and specificity estimates. Second, the accuracy of the scales for identifying people with stroke/TIA may also be affected by confounders such as differences in age, sex, the proportion of patients with ischemic stroke, hemorrhagic stroke or TIA (Table 1), the difference in inclusion criteria between studies and in the provider applying the scale. In most studies, the stroke scale assessment was performed by paramedics or nurses, making the evidence indirect for the first aid setting. However, Liferidge et al. found that lay providers were able to use the CPSS to detect stroke in volunteers with simulated stroke with 94.3% sensitivity (95% CI [86.6-100.0]) and 82.93% specificity (95% CI [70.4-95.3]) [40]. Third, the overall Kappa for the review of titles/abstracts was moderate (Kappa = 0.44). This reflects difficulties in correctly identifying observational studies of stroke recognition in adults in a prehospital setting. However, based on a subsequent review of reference lists, we did not identify any additional articles that were missed during the review process. Finally, the risk of bias is serious or moderate in four of six studies due to possible confounding, missing data and the different time interval definitions for the outcome “time to treatment” [20, 29, 33, 34]. Risk of bias is high or unclear in most of the diagnostic studies for patient selection or quality of the reference standard, and most of the studies failed to include all eligible consecutive participants. The methodology used by the studies is often different, measurement of the time to treatment is not the same, and the method and the length of training used to teach the score varied between studies. There is a high level of between-study heterogeneity, and therefore we must interpret the summary estimate result with caution.

## Conclusions

The use of stroke recognition scales in the prehospital setting should be encouraged. They assist in the detection of the presence of stroke and reduce the time from symptom onset to definitive treatment.

There are many stroke scales available for use in the prehospital environments and the selection of which scale to use remains complex. This review has shown that the use of the FAST and OPSS stroke recognition scales increases the proportion of stroke patients who receive therapy in the first hours following the onset of stroke. Furthermore, FAST and MASS are the scales with the highest sensitivity, while CPSS is the scale with the highest specificity. When blood glucose measurement is possible in the prehospital setting, LAPSS and MASS are scales with sensitivities similar to that for CPSS and FAST but provide greater specificity for the recognition of stroke.

## Appendices

APPENDIX A: Full search strategy for each database

2015 ILCOR FATF CoSTR Systematic Review on Stroke Recognition

The results of the search strategy for the 2015 ILCOR First Aid Task Force (FATF) systematic review on stroke recognition are presented in Table 6.

**Table 6:** Results of the search for strategy, 2015 ILCOR FATF systematic review on stroke recognition

Databases Searched	Date of Search	Number of Articles
All Medline <1946 - 2019>	September 26, 2019	2098
All Embase <1947 - 2019>	September 26, 2019	1316
Cochrane Trials only <1947 - 2019>	September 28, 2019	30
	Total (<1947 – 2019)	3759

After duplicates were removed, title and abstract were screened, full-text articles were independently assessed and disagreements resolved through discussion, 24 articles were included in the 2015 systematic review.

Rerun Strategy from January 2014 to September 2019

The rerun strategy from January 2014 to September 2019 in three databases (MEDLINE, EMBASE and COCHRANE) and results are presented in Tables 7, 8 (Date of search: 26/09/2019).

**Table 7:** Rerun strategy from January 2014 to September 2019 in MEDLINE, EMBASE and COCHRANE (date of search: 26/09/2019)

#	Searches	Number of Articles
Database(s): MEDLINE(R), via the PUBMED interface		
1	Stroke[MeSH Terms]	125,634
2	acute[Title/Abstract]	1,133,443
3	#1 AND #2	30,212
4	acute stroke*[Title/Abstract]	14,610
5	acute cerebrovascular accident*[Title/Abstract]	200
6	#3 OR #4 OR #5	34,856
7	scale*[Title/Abstract]	768,820
8	score*[Title/Abstract]	855,723
9	scoring[Title/Abstract]	75,964
10	OR #7-#9	1,445,022
11	Time-to-Treatment[MeSH Terms]	5308
12	©[MeSH Terms]	1,162,338
13	time-to-treatment[Title/Abstract]	3325
14	recogn*[Title/Abstract]	723,108
15	cognitive knowledge[Title/Abstract]	222
16	neurologic outcome*[Title/Abstract]	3587
17	neurologic status[Title/Abstract]	1837
18	OR #11-#17	1,875,735
19	#6 AND #10 AND #18	2185
20	animals[mh] NOT humans[mh]	4,622,905
21	"letter"[pt] OR "comment"[pt] OR "editorial"[pt] or Case Reports[ptyp]	3,603,162
22	#19 NOT #20 NOT #21	2098
23	"2014/01/01"[PDAT] : "2019/09/26"[PDAT]	6,592,744
24	#22 AND #23	1042
Database(s): EMBASE (R), via Embase.com		
1	'cerebrovascular accident'/de	308,474
2	acute:ab,ti	1,585,164
3	1 AND 2	66,054
4	(acute near/3 stroke*):ab,ti	54,551
5	'acute cerebrovascular accident':ab,ti	230
6	'acute cerebrovascular accidents':ab,ti	138
7	#3 AND #4 AND #5 AND #6	86,881
8	'scoring system'/de	246,463
9	'rating scale'/de	108,517
10	scale*:ab,ti	1,008,513
11	score*:ab,ti	1,340,709
12	scoring:ab,ti	117,138
13	OR #8-#12	2,138,593
14	'time to treatment'/de	14,751
15	'time factors'/de	29,856
16	'time to treatment':ab,ti	6073
17	recogn*:ab,ti	914,845
18	'cognitive knowledge':ab,ti	279
19	'neurologic outcome':ab,ti	3760
20	'neurologic outcomes':ab,ti	1836
21	'neurologic status':ab,ti	2500
22	OR #14-#21	968,595
23	#7 AND #13 AND #22	1477
24	animal'/exp NOT 'human'/exp	5,326,301
25	#23 NOT #24	1460
26	[editorial]/lim OR [letter]/lim OR 'case report'/de	4,003,436
27	#25 NOT #26	1408
28	#27 AND [embase]/lim	1316
29	#28 AND (2014-2019)/py	775
Database(s): The Cochrane Library(R)		
1	[mh Stroke]	8451
2	acute:ab,ti	122,671
3	#1 AND #2	2412
4	(acute near/3 stroke*):ab,ti	8484
5	"acute cerebrovascular accident*":ab,ti	8
6	#3 OR #4 OR #5	9134
7	scale*:ab,ti	150,761
8	score*:ab,ti	220,166
9	scoring:ab,ti	10,216
10	#7 OR #8 OR #9	298,259
11	[mh Time-to-Treatment]	264
12	[mh "Time Factors"]	63,000
13	"time-to-treatment":ab,ti	1889
14	recogn*:ab,ti	17,748
15	"cognitive knowledge":ab,ti	33
16	"neurologic outcome*":ab,ti	316
17	"neurologic status":ab,ti	130
18	#11 OR #12 OR #13 OR #14 OR #15 OR #16 OR #17	82,461
19	#6 AND #10 AND #18	345
	from Jan 2014 to Sep 2019	196

**Table 8:** Result of the re-run of search strategy from 1 January 2014 to 26 September 2019

Databases Searched	Date of Search	Number of Results
All Medline <2014 - 2019>	September 26, 2019	1042
All Embase <2014 - 2019>	September 26, 2019	775
Cochrane Trials only <2014 - 2019>	September 28, 2019	196
	Total (<2014 - 2019)	2013
	Total <2014 - 2019 (after deduplication)	1651
	Total after title and abstract screen	40
	Total after full text stage	4

Rerun Strategy from September 2019 to May 2020

Results of the search strategy from September 2019 to May 2020 are presented in Tables 9, 10 (date of search 05/20/2020).

**Table 9:** Results of the search strategy from September 2019 to May 2020 (date of search 05/20/2020)

#	Searches	Number of Articles
Database: MEDLINE(R), via the PUBMED interface		
1	Stroke[MeSH Terms]	1982
2	acute[Title/Abstract]	670
3	#1 AND #2	670
4	acute stroke*[Title/Abstract]	873
5	acute cerebrovascular accident*[Title/Abstract]	4
6	#3 OR #4 OR #5	1351
7	scale*[Title/Abstract]	53,910
8	score*[Title/Abstract]	65,364
9	scoring[Title/Abstract]	4852
10	OR #7-#9	104,908
11	Time-to-Treatment[MeSH Terms]	342
12	"Time Factors" [MeSH Terms]	5072
13	time-to-treatment[Title/Abstract]	244
14	recogn*[Title/Abstract]	30,991
15	cognitive knowledge[Title/Abstract]	10
16	neurologic outcome*[Title/Abstract]	197
17	neurologic status[Title/Abstract]	71
18	OR #11-#17	36,660
19	#6 AND #10 AND #18	65
20	animals[mh] NOT humans[mh]	27,043
21	"letter"[pt] OR "comment"[pt] OR "editorial"[pt] or Case Reports[ptyp]	88,312
22	#19 NOT #20 NOT #21	65
23	"2014/01/01"[PDAT] : "2019/09/26"[PDAT]	
24	#22 AND #23	65
Database(s): EMBASE (R), via Embase.com		
1	'cerebrovascular accident'/de	19,122
2	acute:ab,ti	64,060
3	1 AND 2	4529
4	(acute near/3 stroke*):ab,ti	2787
5	'acute cerebrovascular accident':ab,ti	19
6	'acute cerebrovascular accidents':ab,ti	5
7	#3 OR #4 OR #5 OR #6	5234
8	'scoring system'/de	10,010
9	'rating scale'/de	2474
10	scale*:ab,ti	67,006
11	score*:ab,ti	86,475
12	scoring:ab,ti	6542
13	OR #8-#12	137,402
14	'time to treatment'/de	1767
15	'time factors'/de	3193
16	'time to treatment':ab,ti	467
17	recogn*:ab,ti	37,759
18	'cognitive knowledge':ab,ti	7
19	'neurologic outcome':ab,ti	159
20	'neurologic outcomes':ab,ti	153
21	'neurologic status':ab,ti	86
22	OR #14-#21	42,985
23	#7 AND #13 AND #22	97
24	'animal'/exp NOT 'human'/exp	111,646
25	#23 NOT #24	96
26	[editorial]/lim OR [letter]/lim OR 'case report'/de	134,589
27	#25 NOT #26	86
28	#27 AND [embase]/lim	75
29	#28 AND (2014-2019)/py (from 27/09/2019 to 25/05/2020 for rerun 2020)	93
Database(s): The Cochrane Library(R)		
1	[mh Stroke]	918
2	acute:ab,ti	3664
3	#1 AND #2	316
4	(acute near/3 stroke*):ab,ti	105
5	"acute cerebrovascular accident*":ab,ti	0
6	#3 OR #4 OR #5	173
7	scale*:ab,ti	9795
8	score*:ab,ti	10,794
9	scoring:ab,ti	152
10	#7 OR #8 OR #9	15,526
11	[mh Time-to-Treatment]	44
12	[mh "Time Factors"]	746
13	"time-to-treatment":ab,ti	95
14	recogn*:ab,ti	1890
15	"cognitive knowledge":ab,ti	2
16	"neurologic outcome*":ab,ti	0
17	"neurologic status":ab,ti	0
18	#11 OR #12 OR #13 OR #14 OR #15 OR #16 OR #17	643
19	#6 AND #10 AND #18	21
	from September 2019 to May 2020	23

**Table 10:** Results of the rerun search strategy from 26 September 2019 to 25 May 2020

Databases Searched	Date of Search	Number of Articles
All Medline	May 25, 2020, 2019	65
All Embase < Sept. 2019 to May 2020>	May 25, 2020, 2019	94
Cochrane Trials only < Sept. 2019 to May 2020>	May 25, 2020, 2019	22
	Total (Sept. 2019 to May 2020)	181
	Total < Sept. 2019 to May 2020 (after deduplication)	163
	Total after title and abstract screen	6
	Total after full text stage	0

Global Search Strategy from Inception to May 2020

The results of the global search strategy are presented in Table 11.

**Table 11:** Result of the global search strategy for stroke recognition

Sources	Number of Articles	Number of Articles Selected
2015 ILCOR FATF systematic review on stroke recognition	24	16
Other sources	8	4
2019 rerun search strategy ILCOR FATF	1651	4
2020 rerun search strategy ILCOR FATF	163	0
	Total of included studies	24

APPENDIX B: Characteristics of excluded studies

The characteristics of excluded studies are presented in Table 12.

**Table 12:** Characteristics of excluded studies

First Author, Year	Reasons for Exclusion
Studies from 2014 to September 2019	
Antonenko, 2014	Congress presentation, abstract only, wrong population
Atsumi, 2015	No comparison, effect of a protocol over time
Bergman, 2015	Congress presentation, abstract only
Brininger, 2018	Congress presentation, abstract only
Bugge, 2019	Congress presentation, abstract only
Chen, 2015	Wrong intervention, wrong population
Chen, 2016	Wrong intervention
Ciobanu, 2017	No text found, congress presentation
Dami, 2017	Wrong intervention, stroke recognition by dispatchers
Glidden, 2019	Congress presentation, abstract only, wrong intervention: nursing triage process using the acronym "FLASHED"
Gramling, 2014	Wrong population (children)
Gropen, 2019	Wrong population (large vessel occlusion)
Hamm, 2015	Congress presentation, abstract only
He, 2017	Scale use by GPs
Hsieh, 2016	Wrong scale (assessment of prenotification protocol not only CPSS and they add glycaemia)
Huang, 2016	No specific scale assesses but they assess different various measures taken to reduce delay
Jia, 2017	Wrong intervention, stroke recognition by EMS dispatcher and crew
Jain, 2014	Wrong intervention, scale completed in an Emergency Department
Kaps, 2014	Assessment of the frequency of warning signs in younger patients with stroke with a special regard to FAST
Kharinotova, 2018	Congress presentation, abstract only
Kim, 2016	Congress presentation, abstract only
Lee, 2014	Congress presentation, abstract only, wrong population (in an emergency department)
Mao, 2016	Wrong population (suspected stroke presenting in the emergency department with symptoms or signs within 7 days; Scale completed in an emergency department)
Mould-Millman, 2018	Wrong intervention (assessment of a protocol made by the dispatcher, paramedics and ED)
Neville, 2016	Wrong population (children)
Noorian, 2018	Wrong population (suspicion of stroke with a large vessel occlusion)
Ocstema, 2018	Wrong population (suspicion of stroke limited to posterior circulation stroke)
Ocstema, 2015	Wrong population (limited to ischemic stroke)
Paden, 2015	Congress presentation, abstract only
Purrucker, 2017	Wrong population (suspicion of stroke with a large vessel occlusion)
Quenardelle, 2015	Congress presentation, abstract only
Silva, 2015	Congress presentation, abstract only
Taqi, 2015	Wrong intervention (Large Vessel Occlusion Screening Tool)
Whiteley, 2011	Wrong intervention (scale made in the emergency department)
Zaidi, 2017	Wrong intervention (Large Vessel Occlusion Screening Tool)
Zhai, 2017	Congress presentation, abstract only
Zhao, 2018	Wrong intervention (Large Vessel Occlusion Screening Tool)
Zohrevandi, 2015	Wrong intervention (scale assessed in an Emergency Department)
Studies excluded from 2015 CoSTR ILCOR FATF	
Buck, 2009	Wrong intervention (emergency medical dispatcher)
De Lucas, 2013	Wrong intervention (emergency medical dispatcher)
Jiang, 2014	Wrong intervention (the original research purpose was to validate the ROSIER score in the emergency room and not in prehospital settings)
Kleindorfer, 2007	Exclusion (retrospectively collection of signs by nurses in medical records of all stroke patients)
You, 2013	Wrong population (thrombolytic candidates in acute ischemic stroke only). Exclusion (the aim of this study is to investigate the usefulness of the CPSS to determine stroke severity by comparing CPSS and NIHSS scores in patients who may be candidates for thrombolysis on arrival at the hospital within 6 hours of symptom onset)
Nazliel, 2008	Wrong population (the aim of the study is to determine whether LAMS scores can predict the presence of large vessel occlusions in acute cerebral ischemia patients)
Nor, 2005	Wrong intervention (emergency physicians with ROSIER and retrospective calculation based on neurologist-recorded signs for CPSS, LAPSS and FAST)
Whiteley, 2011	Wrong intervention (The stroke scale was completed by emergency physicians)
Yock-Corrales, 2011	Wrong population and exclusion criteria (scale applied retrospectively to children only with ischemic stroke)
Studies excluded from rerun search strategy from 2019 to 2020	
Kaps, 2014	Wrong population (younger people), wrong intervention (retrospective analysis on hospital signs)
Willaert, 2020	Congress presentation, abstract only
Colton, 2020	Wrong intervention, wrong population (Intracranial hemorrhage only)
Car, 2020	Wrong intervention (emergent large vessel occlusion)
Madhok, 2019	Wrong intervention (no limit to CPSS but a whole protocol before and after the implementation)
Lee, 2020	Wrong intervention

APPENDIX C: Evidence profile tables

Evidence Profile Tables for Observational Studies

Evidence profile tables for observational studies are presented in Tables 13-18.

**Table 13:** Evidence profile table for Kurashiki Prehospital Stroke Scale (KPSS) CI: Confidence interval; RR: Risk ratio; MD: Mean difference Question: KPSS compared to Standard assessment for Adults with suspected acute stroke Bibliography: Iguchi 2011 [34] Explanations: a- The score was not calculated for 174 patients in the series, small sample size; b- The KPSS is used to identify thrombolytic candidates; c- Selective reported result.

Certainty Assessment							No. of Patients		Effect		Certainty	Importance
No. of studies	Study design	Risk of bias	Inconsistency	Indirectness	Imprecision	Other considerations	KPSS	Standard assessment	Relative (95% CI)	Absolute (95% CI)		
Rate of patient admitted <3 h with stroke diagnosis												
1	observational studies	very serious ^a^	not serious	serious ^b^	not serious	none	161/256 (62.9%)	91/174 (52.3%)	RR 1.20 (1.01 to 1.43)	105 more per 1 000 (from 5 more to 225 more)	⨁◯◯◯ VERY LOW	CRITICAL
No. of patients who received tPA												
1	observational studies	very serious ^a^	not serious	serious ^b^	not serious	none	35/256 (13.7%)	25/174 (14.4%)	RR 0.95 (0.59 to 1.53)	7 fewer per 1 000 (from 59 fewer to 76 more)	⨁◯◯◯ VERY LOW	IMPORTANT
Onset to admission												
1	observational studies	very serious ^a,c^	not serious	serious ^b^	not serious	none	256	174	The mean onset to admission was 0	MD 0.6 lower (0.83 Lower to 0.37 lower)	⨁◯◯◯ VERY LOW	CRITICAL

**Table 14:** Evidence profile table for Los Angeles Prehospital Stroke Scale (LAPSS) CI: Confidence interval; RR: Risk ratio; MD: Mean difference; OR: Odds ratio; IV TPA: Intravenous tissue Plasminogen Activator Question: LAPSS compared to Standard assessment for Adults with suspected acute stroke Bibliography: Wojner-Alexandrov, 2005 [24] Explanations: a- Downgrade for serious risk of bias for selection of participants; b- The LAPSS is used for paramedics’ decision. The assessment is not limited only to the LAPSS.

Certainty Assessment							No. of Patients		Effect		Certainty	Importance
No. of studies	Study design	Risk of bias	Inconsistency	Indirectness	Imprecision	Other considerations	LAPSS	Standard assessment	Relative (95% CI)	Absolute (95% CI)		
Rate onset to admission < 2 h												
1	observational studies	serious ^a^	not serious	serious ^b^	not serious	none	418/674 (62.0%)	210/362 (58.0%)	RR 1.07 (0.96 to 1.19)	41 more per 1 000 (from 23 fewer to 110 more)	⨁◯◯◯ VERY LOW	CRITICAL
Onset to ED Arrival												
1	observational studies	serious ^a^	not serious	serious ^b^	not serious	none	680	359	-	MD 132 higher (14.68 higher to 249.32 higher)	⨁◯◯◯ VERY LOW	CRITICAL
Treatment with IV tPA of confirmed stroke cases												
1	observational studies	serious ^a^	not serious	serious ^b^	not serious	none	64/533 (12.0%)	21/198 (10.6%)	RR 1.13 (0.71 to 1.80)	14 more per 1 000 (from 31 fewer to 85 more)	⨁◯◯◯ VERY LOW	IMPORTANT
Nb of good diagnosis by paramedics at discharge												
1	observational studies	serious ^a^	not serious	serious ^b^	not serious	none	709/895 (79.2%)	198/323 (61.3%)	RR 1.29 (1.18 to 1.42)	178 more per 1 000 (from 110 more to 257 more)	⨁◯◯◯ VERY LOW	IMPORTANT

**Table 15:** Evidence profile table for Ontario Prehospital Stroke Scale (OPSS) CI: Confidence interval; RR: Risk ratio; OR: Odds ratio; IV TPA: Intravenous tissue Plasminogen Activator Question: OPSST compared with standard assessment for adults with suspected acute stroke Bibliography: Chenkin, 2009 [29] Explanations: a- Very serious risk of bias due to deviation from intended interventions, missing data and confounding factor

Certainty Assessment							No. of Patients		Effect		Certainty	Importance
No. of studies	Study design	Risk of bias	Inconsistency	Indirectness	Imprecision	Other considerations	OPSS	Standard assessment	Relative (95% CI)	Absolute (95% CI)		
Ischemic stroke patients arriving <3 hours												
1	observational studies	very serious ^a^	not serious	not serious	not serious	none	178/554 (32.1%)	69/307 (22.5%)	RR 1.43 (1.12 to 1.82)	97 more per 1 000 (from 27 more to 184 more)	⨁◯◯◯ VERY LOW	CRITICAL
Rate of tPA administration (all patients)												
1	observational studies	very serious ^a^	not serious	not serious	not serious	none	56/554 (10.1%)	18/307 (5.9%)	RR 1.72 (1.03 to 2.88)	42 more per 1 000 (from 2 more to 110 more)	⨁◯◯◯ VERY LOW	IMPORTANT
Diagnosis ischemic stroke												
1	observational studies	very serious ^a^	not serious	not serious	not serious	none	290/554 (52.3%)	145/307 (47.2%)	RR 1,11 (0.96 to 1.28)	52 more per 1 000 (from 19 fewer to 132 more)	⨁◯◯◯ VERY LOW	IMPORTANT

**Table 16:** Evidence profile table for Face, Arm, Speech Time, Emergency Response Protocol (FASTER) CI: Confidence interval; MD: Mean difference; RR: Risk ratio Explanations: a- Very serious risk of bias due to confounding and selection for the reported result; b- Serious imprecision due to incomplete data reporting Question: FASTER compared to Standard assessment for Adults with suspected acute stroke Bibliography: O’Brien, 2012 [20]

Certainty Assessment							No. of Patients		Effect		Certainty	Importance
No. of studies	Study design	Risk of bias	Inconsistency	Indirectness	Imprecision	Other considerations	FASTER	Standard assessment	Relative (95% CI)	Absolute (95% CI)		
Symptom onset to treatment time												
1	observational studies	very serious ^a^	not serious	not serious	serious ^b^	none	17	17	-	MD 32 min fewer (53 fewer to 11 fewer)	⨁◯◯◯ VERY LOW	CRITICAL
Door to CT time												
1	observational studies	very serious ^a^	not serious	not serious	serious ^b^	none	17	17	-	MD 30 min fewer (50 fewer to 11 fewer)	⨁◯◯◯ VERY LOW	CRITICAL
Door to needle time												
1	observational studies	very serious ^a^	not serious	not serious	serious ^b^	none	17	17	-	MD 46 min fewer (71 fewer to 21 fewer)	⨁◯◯◯ VERY LOW	CRITICAL
Onset to door												
1	observational studies	very serious ^a^	not serious	not serious	serious ^b^	none	17	17	-	MD 17 min more (7.3 fewer to 41 more )	⨁◯◯◯ VERY LOW	CRITICAL
Rate of thrombolytic therapy												
1	observational studies	very serious ^a^	not serious	not serious	serious ^b^	none	22/115 (19.1%)	5/67 (7.5%)	RR 2.56 (1.02 to 6.45)	116 more per 1 000 (from 1 more to 407 more)	⨁◯◯◯ VERY LOW	IMPORTANT

**Table 17:** Evidence profile table for Face, Arm, Speech, Time to Call (FAST) Scale CI: Confidence interval; RR: Risk ratio Question: FAST compared to Standard assessment for Adults with suspected acute stroke Bibliography: Harbison, 2003 [33] Explanations: a- Fast is integrated in a specific protocol call “rapid ambulance protocol” and compared with PCDs and ED doctor’s diagnosis of stroke. No information about confounding factors.

Certainty Assessment							No. of Patients		Effect		Certainty	Importance
No. of studies	Study design	Risk of bias	Inconsistency	Indirectness	Imprecision	Other considerations	FAST	Standard assessment	Relative (95% CI)	Absolute (95% CI)		
Rate of patients admitted <3 h with stroke diagnosis												
1	observational studies	very serious ^a^	not serious	not serious	not serious	none	66/137 (48.2%)	32/219 (14.6%)	RR 3.30 (2.29 to 4.75)	336 more per 1 000 (from 188 more to 548 more)	⨁◯◯◯ VERY LOW	CRITICAL

**Table 18:** Evidence profile table for increased public/layperson recognition of signs of stroke CI: Confidence interval; RR: Risk ratio; OR: Odds ratio Question: Knowledge on FAST symptoms Pretest and Posttest Survey Results Bibliography: Wall, 2008 [23] Explanations: a- Only one study is about cognitive knowledge. This research identifies messages with evidence-based effectiveness for communicating stroke signs and symptoms. The population is limited to non-Hispanic white and non-Hispanic black women aged 40 to 64 years.

Certainty Assessment							No. of Patients		Effect		Certainty	Importance
No. of studies	Study design	Risk of bias	Inconsistency	Indirectness	Imprecision	Other considerations	Knowledge on FAST symptoms	Placebo	Relative (95% CI)	Absolute (95% CI)		
Before education and immediately after												
1	observational studies	not serious	not serious	not serious	serious ^a^	none	68/72 (94.4%)	55/72 (76.4%)	RR 1.24 (1.07 to 1.42)	183 more per 1 000 (from 53 more to 213 more)	⨁⨁⨁◯ MODERATE	
After education and 3 months after												
1	observational studies	not serious	not serious	not serious	serious ^a^	none	63/65 (96.9%)	64/65 (98.5%)	RR 0.98 (0.93 to 1.04)	20 fewer per 1 000 (from 69 fewer to 39 more)	⨁⨁⨁◯ MODERATE	

Evidence Profile Tables for Diagnosis Studies

Evidence profile tables for diagnosis studies are presented in Tables 19-22.

**Table 19:** Evidence profile table for Face, Arm, Speech, Time to Call (FAST) Scale Question: Should FAST analysis be used to diagnose stroke and TIA in patients suspected of stroke? Bibliography: Berglund, 2014 [27]; Bergs, 2010 [14]; Fothergill, 2013 [17]; Pickham, 2019 [21] Explanations: a- three studies have high risk of bias for patient selection, one has high risk of bias for reference standard, two have moderate risk of bias for reference standard and one has moderate risk of bias for flow and timing; b- One study includes the FAST in a protocol and does not test FAST only (Bergs, 2010), c- Inconsistency is considered as serious due to differences in study cohorts, qualification and training of test administrators, and differences in the reference standard. TIA: Transient ischemic attack

Sensitivity	0.86 (95% CI: 0.69 to 0.94)		Prevalence					52.18%	
Specificity	0.38 (95% CI: 0.16 to 0.66)								
Outcome	No. of studies (No. of patients)	Study design	Factors that may decrease certainty of evidence					Effect per 1 000 patients tested	Test accuracy CoE
			Risk of bias	Indirectness	Inconsistency	Imprecision	Publication bias	pre-test probability of 52.18%	
True positives (patients with stroke and TIA)	4 studies^1-4^ 827 patients	cohort & case-control type studies	serious ^a^	serious ^b^	serious ^c^	not serious	none	449 (360 to 490)	⨁◯◯◯ VERY LOW
False negatives (patients incorrectly classified as not having stroke and TIA)								73 (32 to 162)	
True negatives (patients without stroke and TIA)	4 studies^1-4^ 758 patients	cohort & case-control type studies	serious ^a^	serious ^b^	serious ^c^	not serious	none	182 (77 to 316)	⨁◯◯◯ VERY LOW
False positives (patients incorrectly classified as having stroke and TIA)								296 (162 to 401)	

**Table 20:** Evidence profile table for Los Angeles Prehospital Stroke Scale (LAPSS) Question: Should LAPSS analysis be used to diagnose stroke and TIA in patients suspected of stroke? Bibliography: Asimos, 2014 [26]; Bergs, 2010 [14]; Bray, 2005 [15]; Chen, 2013 [16]; Kidwell, 2000 [18]. Explanations: a- Very serious risk of bias due to high risk for patient selection (4/5) and reference standard, Moderate risk of bias due to reference standard (2/5) and flow and timing (1/5). TIA: Transient ischemic attack

Sensitivity	0.78 (95% CI: 0.75 to 0.81)		Prevalence					66.34%	
Specificity	0.86 (95% CI: 0.67 to 0.95)								
Outcome	No. of studies (No. of patients)	Study design	Factors that may decrease certainty of evidence					Effect per 1 000 patients tested	Test accuracy CoE
			Risk of bias	Indirectness	Inconsistency	Imprecision	Publication bias	Pre-test probability of 66.34%	
True positives (patients with stroke and TIA)	5 studies^1-5^ 1928 patients	Cross-sectional (cohort type accuracy study)	Very serious ^a^	Not serious	Not serious	Not serious	None	559 (537 to 580)	⨁⨁◯◯ LOW
False negatives (patients incorrectly classified as not having stroke and TIA)								157 (136 to 179)	
True negatives (patients without stroke and TIA)	5 studies^1-5^ 764 patients	Cross-sectional (cohort type accuracy study)	Very serious ^a^	Not serious	Not serious	Not serious	None	244 (190 to 270)	⨁⨁◯◯ LOW
False positives (patients incorrectly classified as having stroke and TIA)								40 (14 to 94)	

**Table 21:** Evidence profile table for Cincinnati Prehospital Stroke Scale (CPSS) Question: Should CPSS be used to diagnose stroke and TIA in patients suspected of stroke? Bibliography: Asimos, 2014 [26]; Bergs, 2010 [14]; Bray, 2010 [28]; Bray, 2005 [15]; Frendl, 2009 [31]; Kothari, 1999 [25]; Ramanujam, 2008 [35]; English, 2018 [30]; Kim, 2017 [19]; Studnek, 2013 [36]. Explanations: a- High risk of bias for patient selection (9 studies on 10) and unclear risk of bias for reference standard (8 studies on 10) and for flow and timing (9 studies on 10) TIA: Transient ischemic attack

Sensitivity	0.81 (95% CI: 0.70 to 0.89)		Prevalence					43.45%	
Specificity	0.55 (95% CI: 0.39 to 0.69)								
Outcome	No. of studies (No. of patients)	Study design	Factors that may decrease certainty of evidence					Effect per 1 000 patients tested	Test accuracy CoE
			Risk of bias	Indirectness	Inconsistency	Imprecision	Publication bias	Pre-test probability of 41.41%	
True positives (patients with stroke and TIA)	10 studies^1-10^ 2088 patients	Cross-sectional (cohort type accuracy study)	Very serious ^a^	Not serious	Serious	Not serious	None	352 (304 to 387)	⨁◯◯◯ VERY LOW
False negatives (patients incorrectly classified as not having stroke and TIA)								83 (48 to 131)	
True negatives (patients without stroke and TIA)	10 studies^1-10^ 2812 patients	Cross-sectional (cohort type accuracy study)	Very serious ^a^	Not serious	Serious	Not serious	None	311 (221 to 390)	⨁◯◯◯ VERY LOW
False positives (patients incorrectly classified as having stroke and TIA)								254 (175 to 344)	

**Table 22:** Evidence profile table for Melbourne Ambulance Stroke Scale (MASS) Question: Should MASS be used to diagnose stroke and TIA in patients suspected of stroke? Bibliography: Bergs, 2010 [14]; Bray, 2005 [15]; Bray, 2010 [28] Explanations: a- serious risk of bias due to patient selection and unclear risk of bias due to reference standard and flow and timing TIA: Transient ischemic attack

Sensitivity	0.85 (95% CI: 0.79 to 0.90)		Prevalence					29.66%	
Specificity	0.82 (95% CI: 0.69 to 0.91)								
Outcome	No. of studies (No. of patients)	Study design	Factors that may decrease certainty of evidence					Effect per 1000 patients tested	Test accuracy CoE
			Risk of bias	Indirectness	Inconsistency	Imprecision	Publication bias	Pre-test probability of 29.66%	
True positives (patients with stroke and TIA)	3 studies^1-3^ 291 patients	Cross-sectional (cohort type accuracy study)	Very serious ^a^	Not serious	Not serious	Not serious	None	252 (234 to 267)	⨁⨁◯◯ LOW
False negatives (patients incorrectly classified as not having stroke and TIA)								45 (30 to 63)	
True negatives (patients without stroke and TIA)	3 studies^1-3^ 690 patients	Cross-sectional (cohort type accuracy study)	Very serious ^a^	Not serious	Not serious	Not serious	None	577 (485 to 640)	⨁⨁◯◯ LOW
False positives (patients incorrectly classified as having stroke and TIA)								126 (63 to 218)	

## References

[1] GBD 2015 Mortality and Causes of Death Collaborators (2016). Global, regional, and national life expectancy, all-cause mortality, and cause-specific mortality for 249 causes of death, 1980-2015: a systematic analysis for the Global Burden of Disease Study 2015. Lancet.

[2] Singletary EM, Zideman DA, De Buck ED (2015). Part 9: First aid: 2015 international consensus on first aid science with treatment recommendations. Circulation.

[3] Zideman DA, Singletary EM, De Buck ED (2015). Part 9: First aid: 2015 international consensus on first aid science with treatment recommendations. Resuscitation.

[4] Higgins JP, Altman DG, Gotzsche PC (2011). The Cochrane Collaboration's tool for assessing risk of bias in randomised trials. BMJ.

[5] Moher D, Liberati A, Tetzlaff J, Altman DG (2009). Preferred reporting items for systematic reviews and meta-analyses: the PRISMA statement. BMJ.

[6] McHugh ML (2012). Interrater reliability: the kappa statistic. Biochem Med (Zagreb).

[7] Schunemann HJ, Cuello C, Akl EA (2019). GRADE guidelines: 18. How ROBINS-I and other tools to assess risk of bias in nonrandomized studies should be used to rate the certainty of a body of evidence. J Clin Epidemiol.

[8] Whiting PF, Rutjes AW, Westwood ME (2011). QUADAS-2: a revised tool for the quality assessment of diagnostic accuracy studies. Ann Intern Med.

[9] (2013). GRADE Handbook. https://gdt.gradepro.org/app/handbook/handbook.html.

[10] Schunemann HJ, Mustafa RA, Brozek J (2020). GRADE guidelines: 21 part 2. Test accuracy: inconsistency, imprecision, publication bias, and other domains for rating the certainty of evidence and presenting it in evidence profiles and summary of findings tables. J Clin Epidemiol.

[11] Schunemann HJ, Mustafa RA, Brozek J (2020). GRADE guidelines: 21 part 1. Study design, risk of bias, and indirectness in rating the certainty across a body of evidence for test accuracy. J Clin Epidemiol.

[12] Freeman SC, Kerby CR, Patel A, Cooper NJ, Quinn T, Sutton AJ (2019). Development of an interactive web-based tool to conduct and interrogate meta-analysis of diagnostic test accuracy studies: MetaDTA. BMC Med Res Methodol.

[13] Andsberg G, Esbjornsson M, Olofsson A, Lindgren A, Norrving B, von Euler M (2017). PreHospital ambulance stroke test - pilot study of a novel stroke test. Scand J Trauma Resusc Emerg Med.

[14] Bergs J, Sabbe M, Moons P (2010). Prehospital stroke scales in a Belgian prehospital setting: a pilot study. Eur J Emerg Med.

[15] Bray JE, Martin J, Cooper G, Barger B, Bernard S, Bladin C (2005). Paramedic identification of stroke: community validation of the melbourne ambulance stroke screen. Cerebrovasc Dis.

[16] Chen S, Sun H, Lei Y (2013). Validation of the Los Angeles pre-hospital stroke screen (LAPSS) in a Chinese urban emergency medical service population. PLoS ONE.

[17] Fothergill RT, Williams J, Edwards MJ, Russell IT, Gompertz P (2013). Does use of the recognition of stroke in the emergency room stroke assessment tool enhance stroke recognition by ambulance clinicians?. Stroke.

[18] Kidwell CS, Starkman S, Eckstein M, Weems K, Saver JL (2000). Identifying stroke in the field. Prospective validation of the Los Angeles prehospital stroke screen (LAPSS). Stroke.

[19] Kim DH, Kim SW, Jun SM, Kim JK (2017). Accuracy of prehospital stroke recognition by paramedics and TPA therapy rate after transportation. J Neurol Sci.

[20] O'Brien W, Crimmins D, Donaldson W, Risti R, Clarke TA, Whyte S, Sturm J (2012). FASTER (Face, Arm, Speech, Time, Emergency Response): experience of Central Coast Stroke Services implementation of a pre-hospital notification system for expedient management of acute stroke. J Clin Neurosci.

[21] Pickham D, Valdez A, Demeestere J (2019). Prognostic value of BEFAST vs. FAST to identify stroke in a prehospital setting. Prehosp Emerg Care.

[22] Vanni S, Polidori G, Pepe G, Chiarlone M, Albani A, Pagnanelli A, Grifoni S (2011). Use of biomarkers in triage of patients with suspected stroke. J Emerg Med.

[23] Wall HK, Beagan BM, O'Neill J, Foell KM, Boddie-Willis CL (2008). Addressing stroke signs and symptoms through public education: the Stroke Heroes Act FAST campaign. Prev Chronic Dis.

[24] Wojner-Alexandrov AW, Alexandrov AV, Rodriguez D, Persse D, Grotta JC (2005). Houston paramedic and emergency stroke treatment and outcomes study (HoPSTO). Stroke.

[25] Kothari RU, Pancioli A, Liu T, Brott T, Broderick J (1999). Cincinnati Prehospital Stroke Scale: reproducibility and validity. Ann Emerg Med.

[26] Asimos AW, Ward S, Brice JH, Rosamond WD, Goldstein LB, Studnek J (2014). Out-of-hospital stroke screen accuracy in a state with an emergency medical services protocol for routing patients to acute stroke centers. Ann Emerg Med.

[27] Berglund A, Svensson L, Wahlgren N, von Euler M (2014). Face Arm Speech Time Test use in the prehospital setting, better in the ambulance than in the emergency medical communication center. Cerebrovasc Dis.

[28] Bray JE, Coughlan K, Barger B, Bladin C (2010). Paramedic diagnosis of stroke: examining long-term use of the Melbourne Ambulance Stroke Screen (MASS) in the field. Stroke.

[29] Chenkin J, Gladstone DJ, Verbeek PR, Lindsay P, Fang J, Black SE, Morrison L (2009). Predictive value of the Ontario prehospital stroke screening tool for the identification of patients with acute stroke. Prehosp Emerg Care.

[30] English SW, Rabinstein AA, Mandrekar J, Klaas JP (2018). Rethinking prehospital stroke notification: assessing utility of emergency medical services impression and Cincinnati Prehospital Stroke Scale. J Stroke Cerebrovasc Dis.

[31] Frendl DM, Strauss DG, Underhill BK, Goldstein LB (2009). Lack of impact of paramedic training and use of the Cincinnati prehospital stroke scale on stroke patient identification and on-scene time. Stroke.

[32] Greenberg K, Lesenskyj A, Eichorn D, Maxwell CR, D’Ambrosio M, Vezne-daroglu E, Binning MJ (2017). Change takes time: EMS as the spark plug for faster acute ischemic stroke care. Mathews J Emerg Med.

[33] Harbison J, Hossain O, Jenkinson D, Davis J, Louw SJ, Ford GA (2003). Diagnostic accuracy of stroke referrals from primary care, emergency room physicians, and ambulance staff using the face arm speech test. Stroke.

[34] Iguchi Y, Kimura K, Watanabe M, Shibazaki K, Aoki J (2011). Utility of the Kurashiki Prehospital Stroke Scale for hyperacute stroke. Cerebrovasc Dis.

[35] Ramanujam P, Guluma KZ, Castillo EM (2008). Accuracy of stroke recognition by emergency medical dispatchers and paramedics--San Diego experience. Prehosp Emerg Care.

[36] Studnek JR, Asimos A, Dodds J, Swanson D (2013). Assessing the validity of the Cincinnati Prehospital Stroke Scale and the medic prehospital assessment for code stroke in an urban emergency medical services agency. Prehosp Emerg Care.

[37] Brandler ES, Sharma M, Sinert RH, Levine SR (2014). Prehospital stroke scales in urban environments: a systematic review. Neurology.

[38] Rudd M, Buck D, Ford GA, Price CI (2016). A systematic review of stroke recognition instruments in hospital and prehospital settings. Emerg Med J.

[39] Zhelev Z, Walker G, Henschke N, Fridhandler J, Yip S (2019). Prehospital stroke scales as screening tools for early identification of stroke and transient ischemic attack. Cochrane Database Syst Rev.

[40] Liferidge AT, Brice JH, Overby BA, Evenson KR (2004). Ability of laypersons to use the Cincinnati Prehospital Stroke Scale. Prehosp Emerg Care.

